# Application of ammonium to a N limited arable soil enriches a succession of bacteria typically found in the rhizosphere

**DOI:** 10.1038/s41598-022-07623-4

**Published:** 2022-03-08

**Authors:** Mario Hernández-Guzmán, Valentín Pérez-Hernández, Yendi E. Navarro-Noya, Marco L. Luna-Guido, Nele Verhulst, Bram Govaerts, Luc Dendooven

**Affiliations:** 1 Laboratory of Soil Ecology, CINVESTAV, Av. Instituto Politécnico Nacional 2508, Col. San Pedro Zacatenco, Alcaldía Gustavo A Madero, Mexico City, Mexico; 2 Department of Chemistry and Biochemistry, Instituto Tecnológico de Tuxtla-Gutiérrez, Tuxtla Gutiérrez, Mexico; 3 Centro de Investigación en Ciencias Biológicas, Universidad Autónoma de Tlaxcala, Tlaxcala, México; 4 International Maize and Wheat Improvement Center (CIMMYT), El Batán, Texcoco, Mexico; 5 Cornell University, Ithaca, USA

**Keywords:** Microbiology, Microbial communities, Microbial ecology, Microbiome

## Abstract

Crop residue management and tillage are known to affect the soil bacterial community, but when and which bacterial groups are enriched by application of ammonium in soil under different agricultural practices from a semi-arid ecosystem is still poorly understood. Soil was sampled from a long-term agronomic experiment with conventional tilled beds and crop residue retention (CT treatment), permanent beds with crop residue burned (PBB treatment) or retained (PBC) left unfertilized or fertilized with 300 kg urea-N ha^−1^ and cultivated with wheat (*Triticum durum* L.)/maize (*Zea mays* L.) rotation. Soil samples, fertilized or unfertilized, were amended or not (control) with a solution of (NH_4_)_2_SO_4_ (300 kg N ha^−1^) and were incubated aerobically at 25 ± 2 °C for 56 days, while CO_2_ emission, mineral N and the bacterial community were monitored. Application of NH_4_^+^ significantly increased the C mineralization independent of tillage-residue management or N fertilizer. Oxidation of NH_4_^+^ and NO_2_^−^ was faster in the fertilized soil than in the unfertilized soil. The relative abundance of *Nitrosovibrio*, the sole ammonium oxidizer detected, was higher in the fertilized than in the unfertilized soil; and similarly, that of *Nitrospira*, the sole nitrite oxidizer. Application of NH_4_^+^ enriched *Pseudomonas*, *Flavisolibacter, Enterobacter* and *Pseudoxanthomonas* in the first week and *Rheinheimera*, *Acinetobacter* and *Achromobacter* between day 7 and 28. The application of ammonium to a soil cultivated with wheat and maize enriched a sequence of bacterial genera characterized as rhizospheric and/or endophytic independent of the application of urea, retention or burning of the crop residue, or tillage.

## Introduction

Soil microorganisms are vital in the cycling of carbon (C), nitrogen (N) and phosphorus (P). Nitrogen enters an ecosystem through the conversion of dinitrogen (N_2_) to ammonium (NH_4_^+^) by free living or symbiotic N_2_ fixators microorganisms. When ammonium is available in soil it is oxidized by ammonium oxidizing bacteria and ammonium oxidizing archaea to nitrite (NO_2_^−^) and by nitrite oxidizing bacteria to nitrate (NO_3_^−^). Nitrate, which easily dissolves in water, is readily taken up by plants^1^. Nitrifiers play a central role in N cycling and their activity is controlled by environmental factors, such as NH_4_^+^ availability, pH, salinity, water content, temperature and agricultural practices^2^.

Conventional agricultural practices (CP) in Mexico include generally monoculture and tillage, with large variations in N fertilizer applied. Crop residue is removed often for fodder or burned. These intensive agricultural practices have reduced strongly soil organic matter content and deteriorated most arable soils^3,4^. Soil organic matter is not replenished as crop residues are removed and tillage breaks up aggregates liberating physically protected organic material. The decrease in soil organic matter and a less developed soil structure reduces water infiltration and the absent of soil cover promotes soil and wind erosion. Conservation agriculture (CA), i.e. minimum tillage, crop rotation and retaining some of the crop residue on the soil surface, has been suggested as an alternative to stop the decline in soil fertility and even reverse it^5^. A long-term field trial was started by the "International Maize and Wheat Improvement Center" (CIMMYT) to investigate the effect of different agricultural practices, i.e. tillage and residue management, combined with different N fertilization practices on yields and soil characteristics in the Yaqui Valley (near Ciudad Obregon, Sonora) in north-western Mexico in 1992^6^. In the Yaqui Valley, farmers mainly grow durum wheat (*Triticum durum* L.) in monoculture during the winter season under furrow irrigation and sometimes maize as summer crop^6^. Crop residue is incorporated through tillage or burned.

The field trial had a wheat and maize (*Zea mays* L.) crop rotation. This study used a subset of treatments with two N fertilizer application rates (no fertilizer versus 300 kg urea-N ha^−1^ for wheat) and three tillage-residue managements (tilled bed planting with residue incorporated [CT treatment] and permanent beds with residue burned [PBB treatment] and retained [PBC treatment]). Earlier research suggested that the retention of crop residue might lead to a limited N availability in the soil and this might affect C and N dynamics, grain quality and the bacterial communities^7,8^. High urea fertilization rates affect nitrifiers and ureolytic soil microbial communities^9^. For instance, the application of inorganic fertilization enriched Bacillales in the maize rhizosphere^10^. It accelerated the mineralization of soil organic matter the first days after N fertilizer application^11^ and stimulated nitrification activity in soil (e.g.^12,13^). Therefore, treatments were sampled in triplicate and soil from each treatment (*n* = 6) was amended with 300 mg NH_4_^+^-N in the laboratory or left unamended and incubated aerobically for 56 days, while emissions of CO_2_, dynamics of mineral N and the bacterial community were monitored. The objectives of this study were to determine how the bacterial communities were affected by (i) the tillage-residue management, (ii) the application of inorganic N fertilizer in the field and (iii) the application of NH_4_^+^ in the laboratory. We found that the application of ammonium to an unfertilized or N fertilized soil cultivated with wheat and maize enriched a sequence of bacterial genera characterized as rhizospheric and/or endophytic independent of the agricultural practices applied.

## Results

### Carbon and nitrogen mineralization

**Figure 1:**
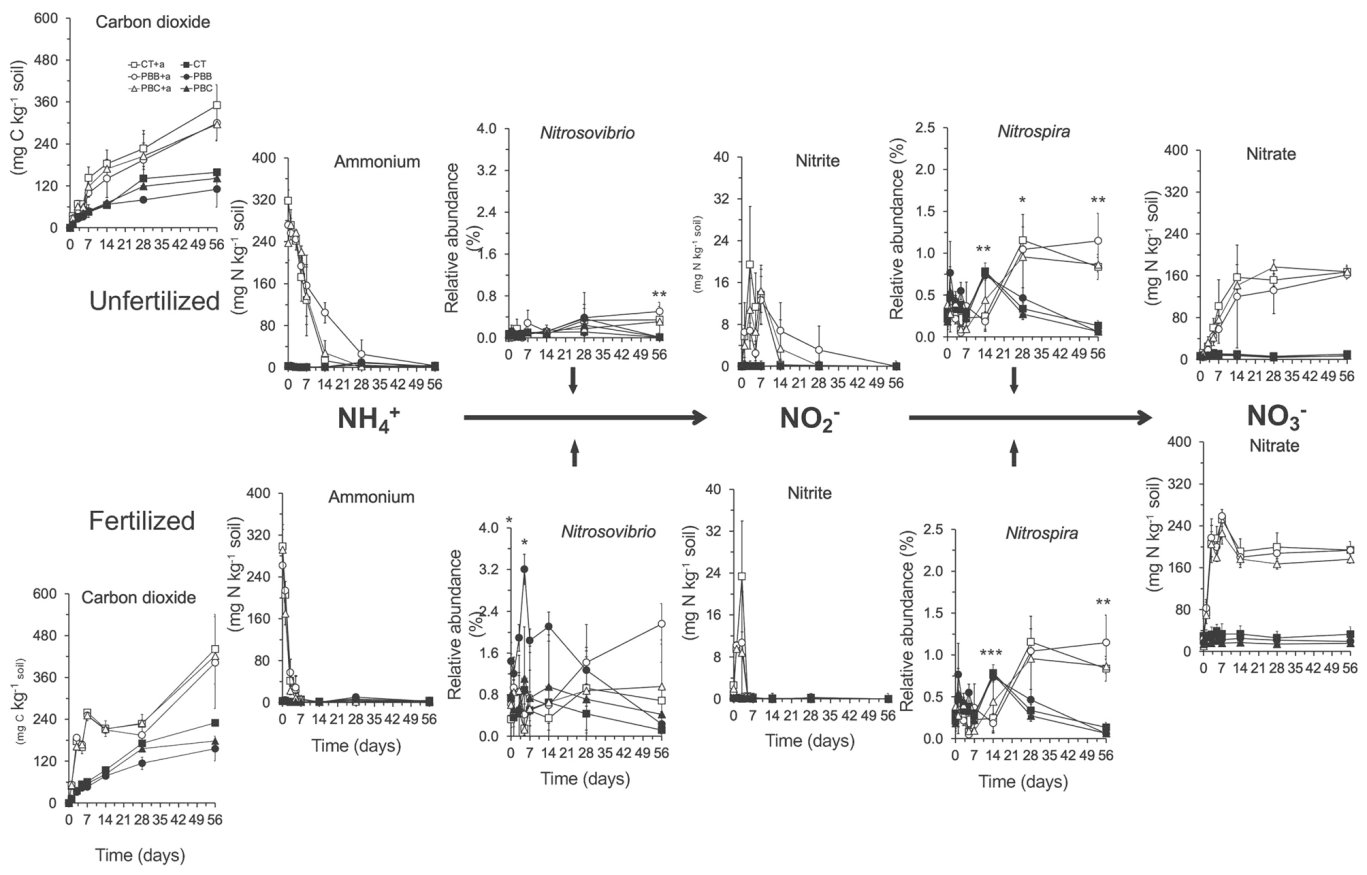
Emission of carbon dioxide (mg C kg^−1^), concentration of ammonium (NH_4_^+^), nitrite and nitrate (mg N kg^−1^ soil), and the relative abundance (%) of the ammonium oxidizer *Nitrosovibrio* and nitrite oxidizer *Nitrospira* in the unfertilized soil and soil fertilized with 300 kg urea-N ha^−1^ with conventional tilled beds and crop residue retained (CT) left unamended (filled black square) or amended with 300 mg NH_4_^+^-N (open square), permanent beds with crop residue burned (PBB) left unamended (filled black circle) or amended with 300 mg NH_4_^+^-N (open circle) and permanent beds with crop residue retained (PBC) incubated left unamended (filled black triangle) or amended with 300 mg NH_4_^+^-N (open triangle) incubated aerobically at 25 ± 2 °C for 56 days. A non-parametric test (aldex.kw function; Kruskal Wallis test) in the ALDEx2 package^14^ was used to determine the effect of application of 300 mg NH_4_^+^-N on the relative abundance of the *Nitrosovibrio* and *Nitrospira* using the centered-log-ratio transformed counts, i.e. clr-transformation, on the different sampling days with ***P < 0.001, **P < 0.01 and *P* ≥ 0.001, and *P < 0.05 *P* ≥ 0.01.

Application of NH_4_^+^ and N fertilizer increased significantly the CO_2_ emitted after 56 days (*P* < 0.05), but tillage-residue management did not affect it (Fig. 1). The amount of NH_4_^+^ in the unamended fertilized and unfertilized soil remained < 10 mg N kg^−1^ soil. The NH_4_^+^-N concentration in the unfertilized soil amended with 300 mg NH_4_^+^-N decreased sharply and was ≤ 18 mg N kg^−1^ soil in the CT and PBC treatments after 28 days, but still 105 mg N kg^−1^ soil in the PBB treatment and significantly higher than in the CT and PBC treatments (*P* < 0.05). The NH_4_^+^-N concentration in the soil fertilized with 300 kg urea-N ha^−1^ and amended with 300 mg NH_4_^+^-N dropped even faster than in the unfertilized and was ≤ 7 mg N kg^−1^ soil after only 7 days.

The concentration of NO_2_^−^ remained < 0.2 mg N kg^−1^ soil in the unamended soil, but application of NH_4_^+^ increased it sharply (Fig. 1). The NO_2_^−^ concentration reached a maximum at day 3 in both the fertilized and unfertilized soil amended with NH_4_^+^ and decreased thereafter, but the decrease was faster in the fertilized than in the unfertilized soil. The concentration of nitrate remained < 13 mg N kg^−1^ soil in the unamended unfertilized soil and < 40 mg N kg^−1^ in the unamended fertilized soil. The application of NH_4_^+^ increased the concentration of nitrate sharply, with the fastest increase found in the fertilized soil. Of the 300 mg NH_4_^+^-N applied, 135 mg N kg^−1^ was not accounted for as NO_2_^−^ or NO_3_^−^ in the unfertilized soil and 122 mg N kg^−1^ in the fertilized soil after 56 days.

### Sequencing results and microbial diversity

A total of 3,243,711 joined paired-end, high quality and chimera-free sequences were obtained from the 288 soil samples. Clustering yielded 78,215 bacterial OTUs based on 97% nucleotide similarity cutoff. The rarefaction curves of the number of OTUs versus the number of sequences per treatment were asymptotic (Fig. S1a). As such, a sufficient sequencing depth was obtained and analyzing more sequences would have yielded only a limited number of more OTUs. The average Good’s coverage was 83%.

Overall, 38 different bacterial phyla, 111 classes, 180 orders, 235 families and 387 genera were detected in the soil. Proteobacteria (relative abundance 51.91%) was the dominant bacterial phylum in soil followed by Acidobacteria (20.48%) and Firmicutes (9.97%) (Fig. S2). *Bacillus* (6.04%) was the dominant bacterial genus followed by *Pseudomonas* (4.49%) and *Halomonas* (4.38%) (Fig. S3).

The bacterial diversity dynamics measured in terms of Hill numbers at *q* = 0, 1 and 2 were similar in the different treatments of the unamended unfertilized and fertilized soil and showed a sharp drop at day 56 compared to day 28 (Fig. S1b). Application of NH_4_^+^ changed the dynamics of the Hill numbers compared to the unamended soil, but there was no significant effect of treatments or application of fertilizer.

### Bacterial community in the unamended and NH_4_^+^-amended soil as affected by tillage-residue management

**Figure 2:**
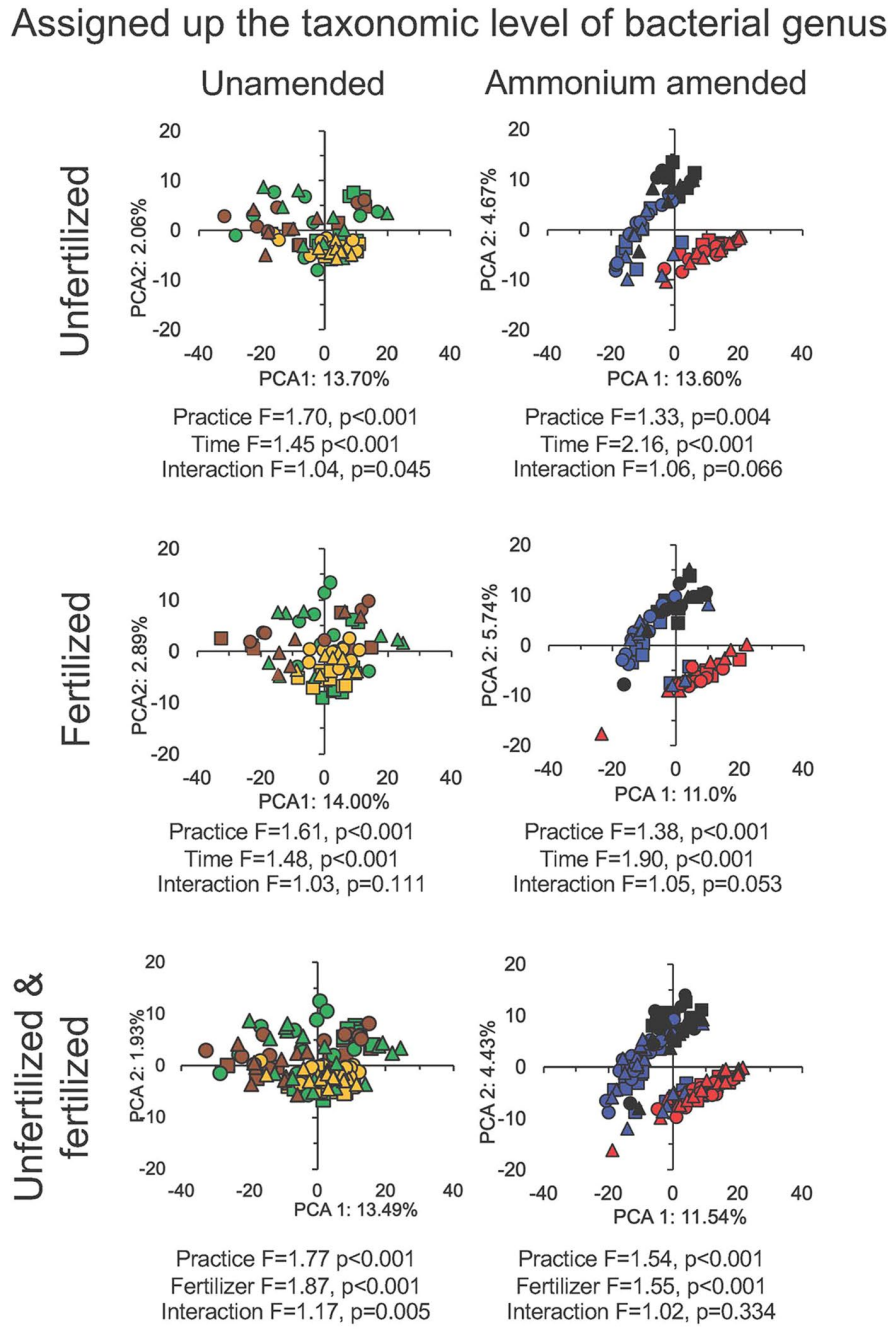
Principal component analysis (PCA) with the converted sequence counts of all bacterial groups classified up to the genus level using the centred log-ratio transformation (aldex.clr argument, ALDEx2 package^14^) in the unfertilized soil or soil fertilized with 300 kg urea-N ha^−1^ left unamended with conventional tilled beds (CT) at day 0, 1 and 3 (filled green square), at day 5, 7 and 14 (filled orange square) and at day 28 and 56 (filled brown square), permanent beds with crop residue burned (PBB) at day 0, 1 and 3 (filled green circle), at day 5, 7 and 14 (filled orange circle) and at day 28 and 56 (filled brown circle) and permanent beds with crop residue retained (PBC) at day 0, 1 and 3 (filled green triangle), at day 5, 7 and 14 (filled orange triangle) and at day 28 and 56 (filled brown triangle) or amended with 300 mg NH_4_^+^-N kg^−1^ dry soil with CT at day 0, 1 and 3 (filled red square), at day 5, 7 and 14 (filled blue square) and at day 28 and 56 (filled black square), PBB at day 0, 1 and 3 (filled red circle), at day 5, 7 and 14 (filled blue circle) and at day 28 and 56 (filled black circle) and PBC at day 0, 1 and 3 (filled red triangle), at day 5, 7 and 14 (filled blue triangle) and at day 28 and 56 (filled black triangle), incubated aerobically at 25 ± 2 °C for 56 days. F and p values were determined with a perMANOVA analysis.

**Figure 3:**
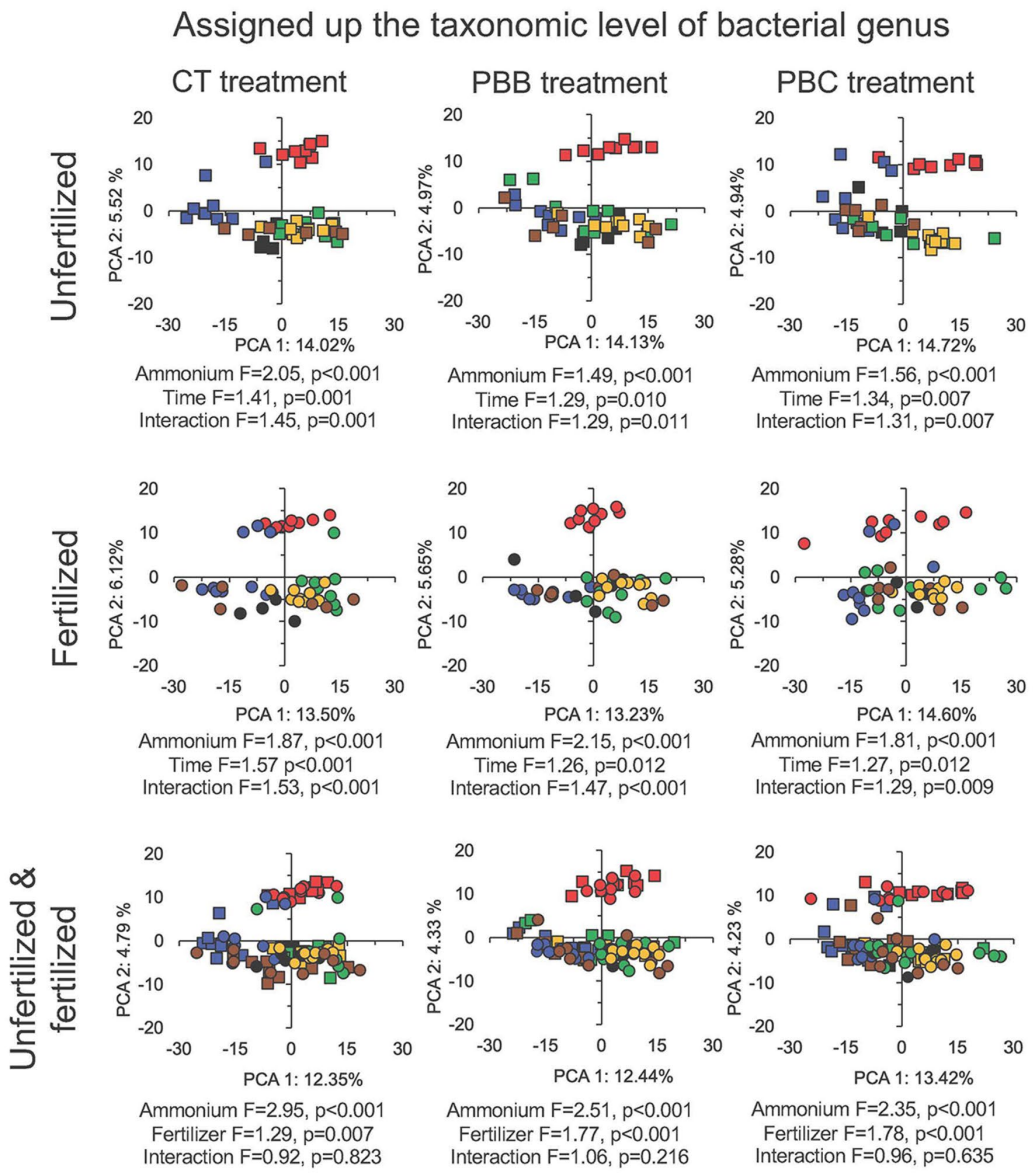
Principal component analysis (PCA) with the converted sequence counts of all bacterial groups classified up to the genus level using the centred log-ratio transformation (aldex.clr argument, ALDEx2 package^14^) in the unfertilized soil left unamended with permanent beds conventional tilled with residue retained (CT treatment) or permanent beds with crop residue burned (PBB treatment) or permanent beds with crop residue retained (PBC treatment) at day 0, 1 and 3 (filled green square), at day 5, 7 and 14 (filled orange square) and at day 28 and 56 (filled brown square), or ammonium-amended at day 0, 1 and 3 (filled red square), at day 5, 7 and 14 (filled blue square) and at day 28 and 56 (filled black square), and in soil fertilized with 300 kg urea-N ha^−1^ left unamended at day 0, 1 and 3 (filled green circle), at day 5, 7 and 14 (filled orange circle) and at day 28 and 56 (filled brown circle) or amended with 300 mg NH_4_^+^-N kg^−1^ at day 0, 1 and 3 (filled red circle), at day 5, 7 and 14 (filled blue circle) and at day 28 and 56 (filled black circle) incubated aerobically at 25 ± 2 °C for 56 days. F and p values were determined with a perMANOVA analysis.

A large number of bacterial groups assigned up to the level of genus was significantly affected by tillage-residue management in the unamended and NH_4_^+^ amended unfertilized and fertilized soil (*P* < 0.05) (Table S1). The PCA showed a clear effect of time on the bacterial community in the NH_4_^+^-amended soil, but not always the effect of tillage-residue management (Figs. 2 and 3). The perMANOVA analysis showed that time and tillage-residue management had a highly significant effect on the bacterial community structure in the fertilized and unfertilized unamended and NH_4_^+^-amended soil (*P* ≤ 0.004, Fig. 2). Additionally, the in-field N fertilizer rate had a highly significant effect on the bacterial community structure in both the unamended and NH_4_^+^-amended soil and affected significantly the relative abundance of a wide range of bacterial groups *(P* < 0.001).

### The bacterial community as affected by the application of NH_4_^+^

**Figure 4:**
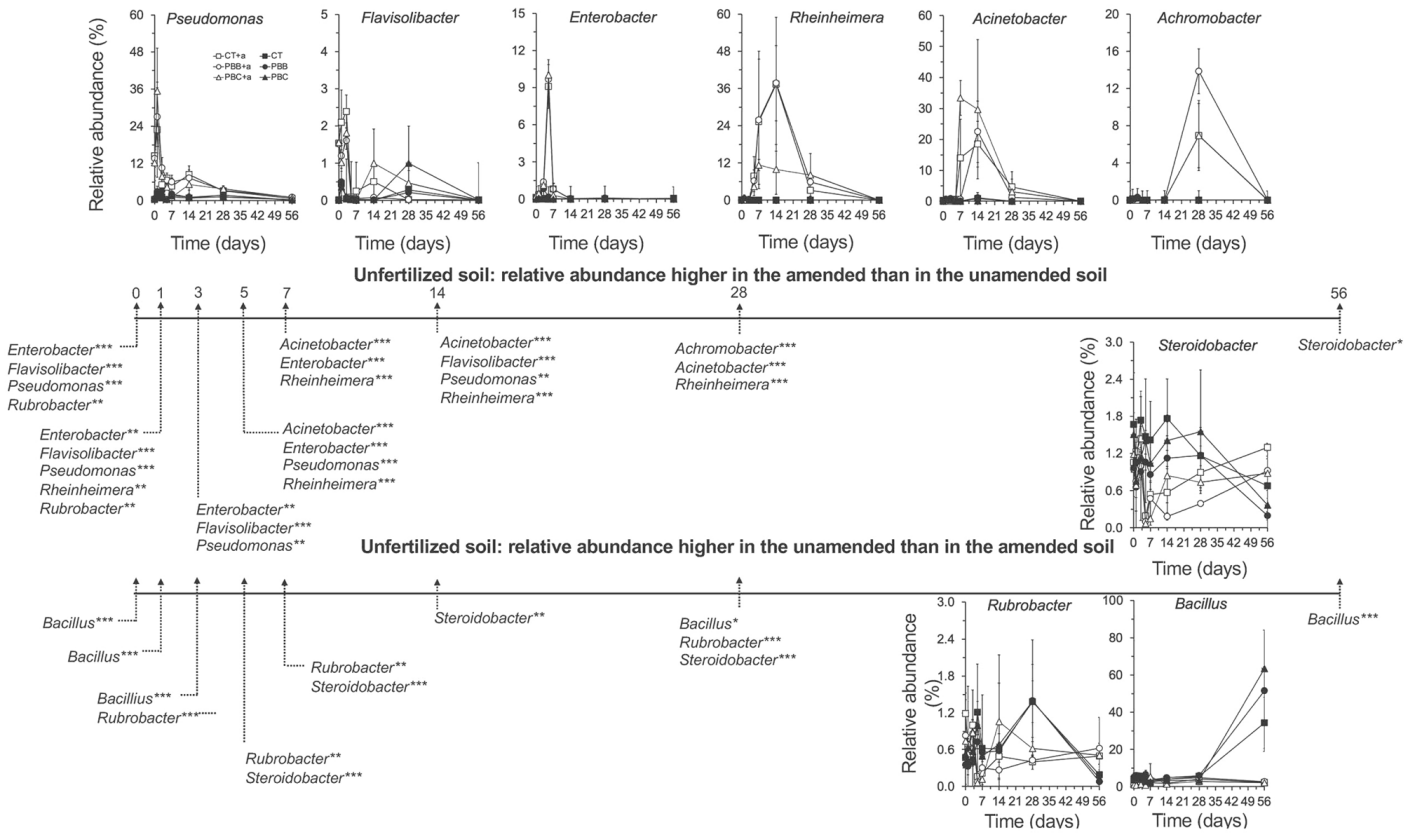
Changes in the relative abundance (%) of bacterial genera in the unfertilized soil with conventional tilled beds and crop residue retained (CT) left unamended (filled black square) or amended with 300 mg NH_4_^+^-N (open square), permanent beds with crop residue burned (PBB) left unamended (filled black circle) or amended with 300 mg NH_4_^+^-N (open circle) and permanent beds with crop retained incubated left unamended (filled black triangle) or amended with 300 mg NH_4_^+^-N (open triangle) incubated aerobically at 25 ± 2 °C for 56 days. A non-parametric test (aldex.kw function; Kruskal Wallis test) in the ALDEx2 package^14^ was used to determine the effect of application of 300 mg NH_4_^+^-N kg^−1^ on the relative abundance of the bacterial genus using the centered-log-ratio transformed counts, i.e. clr-transformation, on the different sampling days with ***P < 0.001, **P < 0.01 and *P* ≥ 0.001, and *P < 0.05 and *P* ≥ 0.01.

Application of NH_4_^+^ to soil had a strong, often immediately and sometimes a long-lasting effect on the relative abundance of a wide range of bacterial groups in the unfertilized and fertilized soil (Figs. 4, S4, S5). The relative abundance of the bacterial groups was affected in a similar way, but the effect was mostly smaller in the fertilized than in the unfertilized soil. Some bacterial groups in the N fertilized soil also responded more rapidly to the application of ammonium so they appeared to be better adapted to N applications than the same bacterial groups in the soil that was not fertilized with urea in the field. A sequence of bacterial genera was enriched highly significantly by the application of NH_4_^+^, compared to the unamended soil in both the fertilized and unfertilized soil, i.e. *Achromobacter, Acinetobacter, Enterobacter, Flavisolibacter*, *Pseudomonas Rheinheimera* (Figs. 4 and S5). Sometimes the relative abundance of the bacterial genus showed a sharp increase on only one day when NH_4_^+^ was applied compared to the unamended soil, e.g. *Achromobacter* on day 28, the effect on others lasted longer, e.g. *Acinetobacter*, *Pseudomonas* and *Rheinheimera*, while still others were sometimes enriched on certain days while on other days their relative abundance decreased, e.g. *Rubrobacter* and *Steroidobacter*. The relative abundance of a wide range of bacterial groups decreased in soil amended with NH_4_^+^. The effect was mostly small except for the enrichment of members of *Bacillus* in the unamended soil on day 56.

The PCA separated the bacterial community in the NH_4_^+^-amended soil from the unamended soil most clearly at day 0, 1 and 3 (Fig. 3). It was only after 28 days that the bacterial community in the NH_4_^+^-amended soil resembled that in the unamended soil. Consequently, the effect of time and application of NH_4_^+^ on the bacterial community was highly significant, but also their interaction (*P* < 0.05). The effect of application of NH_4_^+^ on the bacterial communities was larger than that of the time considering the F values, but it was for a short period of time, from 3 to maximum 5 days (Fig. 3).

Members of only one NH_4_^+^ oxidizer, i.e. *Nitrosovibrio*, and one NO_2_^-^ oxidizer, *Nitrospira,* were detected in the soil (Fig. 1). The relative abundance of *Nitrosovibrio* was significantly higher in the fertilized than in the unfertilized soil, but not that of *Nitrospira* (*P* < 0.001). Treatment had no significant effect on the relative abundance of *Nitrosovibrio* and *Nitrospira* but the application of NH_4_^+^ did on certain days. For instance, members of *Nitrosovibrio* and *Nitrospira* were enriched in the NH_4_^+^-amended soil on day 56.

### Soil functional profiles, and carbon and nitrogen pathway genes

Some clear changes were detected in metabolic processes related to the C and N cycling as determined with FAPROTAX (Figs. S6, S7, S8). Tillage-residue management and application of fertilizer or NH_4_^+^ had a highly significant effect on the carbon and nitrogen pathway genes (Fig. S7). Application of NH_4_^+^ increased the relative abundance of some carbon and nitrogen pathway genes sharply sometimes (Fig. S8). For instance, the relative abundance of the potential ligninolysis showed a large increase in the NH_4_^+^-amended soil compared to the unamended soil on day 0 and 1 but not thereafter, while that of the potential aromatic compound degradation was enriched on days 7, 14 and 28. Application of N fertilizer enriched the potential nitrification, while it was reduced in the NH_4_^+^-amended soil compared to the unamended soil until day 28 but higher on day 56. The nitrite reductase (*nirD* gene, COG2146) and COGs related to transportation and assimilation of N compounds were the most abundant, e.g. glutamine synthetase (COG0174), N-acetylglutamate synthase (COG1246) and ammonium transporter (COG004) (Fig. S9). The perMANOVA results showed that N-cycle related genes were highly significantly affected both by tillage-residue management and NH_4_^+^-N application (*P* < 0.001).

## Discussion

### Carbon and nitrogen mineralization

The soil in the CT, PBB and PBC treatments at CENEB was N depleted, even in the soil fertilized with 300 kg urea-N ha^−1^ for wheat, as application of 300 mg NH_4_^+^-N more than doubled the emitted CO_2_. Previous findings showed that although the retention of crop residues and fertilization increased yield and wheat grain quality it affected N availability in soil^7,8^. As such, the amount of C substrate available for heterotrophs was high but the mineral N severely limited even in the fertilized soil, i.e. C mineralization was impeded by the lack of mineral N so application of 300 mg NH_4_^+^-N stimulated strongly microbial metabolic activity. The composition, e.g. N availability, and the amount of crop residue left in the field will determine its decomposition^15^. The maize and wheat crop residues left in the field had a high C-to-N ratio (approximately 80 for wheat and 57 for maize, USDA Natural Resources Conservation Service (soils.usda.gov/sqi)) so all mineralized N was immobilized by the soil microorganisms. The soil was so N depleted that more than a third of the 300 mg NH_4_^+^ kg^−1^ applied was not accounted for after 56 days. Losses of NH_4_^+^ through abiotic processes, e.g. NH_3_ volatilization, cannot be excluded but were assumed to be small. The soil was only slightly alkaline (pH between 7.7 and 8.1) and the soil was mixed immediately after the ammonium was applied to reduce possible NH_3_ volatilization so most of it was immobilized by the soil microorganisms. The amount of mineral N immobilized in the fertilized soil was similar (i.e. 122 mg NH_4_^+^-N kg^−1^) after 56 days.

### Alpha diversity

The effect of tillage-residue management and crop residue on soil microbial diversity is highly variable and depends on environmental and edaphic factors, and soil nutrient dynamics^16,17^. Some studies found a higher bacterial diversity, i.e. the variability of species, in limited or zero tillage compared to conventional tillage^18^, while others showed little or no effect of tillage on both bacterial richness and diversity^19,20^. In this study, tillage-residue management had no effect on bacterial diversity and richness.

Application of N sources also affects microbial diversity, but their possible effect depends on soil type and fertilizer rates^17,21^. For instance, Staley et al.^22^ studied three urea fertilization regimes on eight different arable soils. They observed only a decrease in soil bacterial diversity at the highest fertilizer application rate, i.e. 500 mg urea-N kg^−1^, and the effect depended on soil characteristics. Fierer et al.^23^, however, found that the bacterial diversity was not affected by N fertilizer application rates as found in this study. However, application of ammonium to the soil increased significantly bacterial richness.

### Soil bacterial community as affected by tillage-residue management

Agricultural practices, such as crop residue management and tillage, are known to affect the bacterial community structure^19,24^. Crop residue management and the composition of the organic material left in the field affects the bacterial community^25^. Retaining crop residue in the field provides heterotrophs with C substrate and enriches copiotrophs. The characteristics of the crop residue, such as its lignin and (hemi)cellulose content and its C-to-N ratio, determine its availability for the soil microorganisms and which of them will be enriched. Kraut-Cohen et al.^26^ reported that even a single tillage event had a significant effect on some microbial groups. Tillage brings crop residue in direct contact with the soil microbial community and breaks up aggregates liberating previously physically protected organic material^27^. These changes in organic material availability will enrich some microorganisms at the expense of others^28^.

Short and long-term inorganic N application did not affect the bacterial community structure in an agricultural soil in Utah^29^, but in this study the effect of N fertilizer application on the bacterial community structure was significant. The length of the experiment, i.e. 20-y, and experimental conditions, such as soil characteristics and agricultural practices, will determine if N fertilizer application will alter the bacterial community structure. In this study, wheat and maize residue, both characterized by a high C-to-N ratio, enriched certain bacteria, such as *Bacillus*, while application of N fertilizer enriched others, such as *Kaistobacter*.

### Soil bacterial community as affected by application of ammonium

After 20-y of cultivating wheat and maize in rotation, a bacterial community had developed adapted to a limited N availability even in the soil fertilized with 300 kg urea-N ha^−1^ for wheat. Application of NH_4_^+^ allowed some bacteria to mineralize the C substrate with a high C-to-N ratio. As a result, the bacterial community structure changed immediately when NH_4_^+^ was applied to soil and was clearly different from the unamended soil. At day 5, the bacterial community structure in the NH_4_^+^-amended soil changed and started to resemble that in the unamended soil so that after 28 days the bacterial community structure in the NH_4_^+^-amended soil was similar to that in the unamended soil.

Application of NH_4_^+^ stimulated the metabolic activity and consecutive growth of specific bacterial groups. Most of these are well known rhizosphere and/or endophytic root bacteria often with plant growth promoting capacity and a copiotrophic lifestyle. Members of *Flavisolibacter* and *Pseudomonas* were the first group of bacteria enriched by the application of ammonium with the latter becoming the dominant bacterial genus at the onset of the experiment. Both genera are considered plant growth promoting rhizobacteria (PGPR)^30,31^. *Pseudomonas* shows rapid growth and can use various substrates as nutrients with a capacity to survive different stress conditions and therefore is a good colonizer of soil^32^. Its members are enriched in the rhizosphere^33^ and have been found abundantly as endophyte in the roots, stems, leaves, pericarp and seeds of tomato plants^34,35^. Most members of *Pseudomonas* have a large metabolic versatility, such as degradation of aromatics and assimilatory N reduction to ammonium^36^, and they can protect plants against pathogens^30^.

Members of *Enterobacter* were the next bacterial genus enriched when NH_4_^+^ was applied to the N depleted soil. *Enterobacter* is also considered a PGPR and has also been found abundantly as endophyte in the roots, stems, leaves, pericarp and seeds of tomato plants^34^. Some strains of *E. cloacae* strains can fix substantial amounts of N_2_ and have other PGPR characteristics^37^. Akita et al.^38^ reported that the strain *E. oligotrophica* could grow in nutrient poor (oligotrophic) medium, but its growth was not affected by high-nutrient medium. Interestingly, Niu et al.^39^ reported that *E. cloacae* was a member of simplified synthetic bacterial community that contained seven strains (also *Pseudomonas putida)* obtained through host-mediated selection of distinctive microbiota assembled from maize roots and it played the role of keystone species in this model ecosystem.

*Rheinheimera, Acinetobacter* and *Pseudoxanthomonas* were the next bacterial genera enriched strongly in the NH_4_^+^-amended soil. Members of *Rheinheimera* (i.e. *R. hassiensis* and *R. muenzenbergensis*) have been detected in the rhizosphere of false rye barley (*Hordeum secalinum* Schreb.)^40^ and it was the dominant bacterial genus in the rhizosphere of hardy sugar cane (*Saccharum arundinaceum* Retz.) grown on organometallic pollutants-rich hazardous distillery sludge^41^. It was also the dominant bacterial genus in the endophyte-enriched root-associated microbiome of rice (*Oryza sativa*) cultivated in soil for 55 days^42^. Members of *Pseudoxanthomonas* are also rhizosphere bacteria^32^ and the strain *P. suwonensis* J1 isolated from soil enriched with rotten leaves and wood has been described as a cellulose-degrading bacterium^43^. Dong et al.^34^ studied the bacterial community in the rootzone, rhizosphere, phyllosphere and endosphere of roots, stems, leaves, fruits and seeds of tomato plants. They found that OTUs belonging to *Pseudomonas* and *Acinetobacter* dominated in the rhizosphere, > 97% of the sequences in the phyllosphere belonged to *Acinetobacter*, and *Acinetobacter*, *Enterobacter* and *Pseudomonas* were the most abundant genera in the roots, stems and leaves. Cordero et al.^44^ studied the bacterial community in rhizosphere and root interior of canola (*Brassica napus* L.), wheat, field pea (*Pisum sativum* L.), and lentil (*Lens culinaris* L.) and found that *Pseudomonas* was one of the dominant genera in the rhizosphere and *Acinetobacter* in the root biome of all these crops.

At day 28, the relative abundance of *Achromobacter*, a salt-tolerant PGPR^45^, was much higher in the NH_4_^+^ amended soil than in the unamended soil. Soares et al.^46^ reported that *A. spanius* B1 an endophyte of the invasive common reed (*Phragmites australis* (Cav.) Trin. ex Steud.) with the capacity to produce indole acetic acid, secrete hydrolytic enzymes, solubilizes phosphate and with antibiosis activity increased common reed growth in soil with a low N content. They suggested that the *A. spanius* presumably increased the growth of common reed by scavenging nitrogenous compounds from the rhizosphere and transferring them to the plant roots.

At day 56, the relative abundance of none of the most abundant bacterial groups showed such a sharp increase as found earlier in the experiment. Contrarily, a range of genera, such as *Steroidobacter* and *Rubrobacter*, were enriched on day 56, although earlier, their relative abundance was often lower in the NH_4_^+^-amended than in the unamended soil. *Steroidobacter* was described by Valverde et al.^47^ as part of a small ‘core’ rhizosphere bacterial community of welwitschia (*Welwitschia mirabilis* Hook.f.). Some members of *Rubrobacter* have been described as oligotrophic which would explain that their relative abundance decreased when NH_4_^+^ was applied to soil^48^.

The relative abundance of a wide range of bacterial groups was reduced when NH_4_^+^ was applied to soil but the effect was small, except for members of *Bacillus* that were enriched strongly in the unamended soil at day 56. Although members of *Bacillus* have been found to be abundant in the rhizosphere of crops, such as maize (e.g. Li et al.^36^), in this study they were not enriched by the application of NH_4_^+^ as other rhizosphere bacteria, such as *Pseudomonas* or *Enterobacter*. The enrichment of phylotypes belonging to *Bacillus* in the unamended soil at day 56 would indicate an oligotrophic lifestyle in the soil studied here.

### Nitrifiers

Only OTUs belonging to one ammonium oxidizing bacteria (AOB) *Nitrosovibrio* and one nitrite oxidizing bacteria (NOB) *Nitrospira* were detected in the soil studied. Shah et al.^49^ reported also *Nitrosovibrio* spp. as the sole AOB and *Nitrosocaldus* spp. as the sole ammonium oxidizing archaea (AOA) when studying the bacterial and archaeal community present in the Pine Barrens Forest of Long Island, NY. Sometimes, more than one AOB and NOB have been detected in soil although both *Nitrosovibrio* and *Nitrospira* are often dominant^29,50^. In this study, the archaeal community was not studied, but AOA are sometimes more important than AOB and might have contributed substantially to the nitrification process in the soil studied (e.g.^51^). The OTUs belonging to the phylum Nitrospirae have a triple-layered cell wall and are enriched under drought stress^52^, conditions predominant in the area with little or no rainfall and high average temperature^53^. *Nitrospira* might also have been benefitted from the lack of nutrients as Liang et al.^54^ reported that application of compost, which is normally nutrient rich, reduced its relative abundance.

Ammonium provides AOA and AOB with the sole energy for growth. Application of urea as inorganic N fertilizer and its subsequent hydrolysis will provide AOB and AOA with energy for growth and the formation of nitrite provides NOB with energy for growth. As such, the relative abundance of *Nitrosospira* was significantly higher in the fertilized than in the unfertilized soil. Interestingly, that was not so for *Nitrospira* although the oxidation of the nitrite as evidenced by dynamics of nitrite and the formation of nitrate was much faster in the first compared to the latter when ammonium was applied to the soil. Ouyang and Norton^29^ reported similar results. They found that application of ammonium sulphate did not affect the abundance of *Nitrospira nxrB* gene determined by real-time quantitative PCR, although ammonium fertilizer application for 4 years significantly increased rates of potential nitrite oxidation determined at 0.15 mM nitrite in soil slurries.

Application of NH_4_^+^ enriched *Nitrosospira* only at the end of the incubation, which would suggest that activity, i.e. oxidation of NH_4_^+^, occurred before growth. The relative abundance of *Nitrospira* was higher in the unamended than in the NH_4_^+^-amended soil at day 14, but enriched in the NH_4_^+^-amended soil at day 28 and 56. This might indicate that oxidation of NO_2_^−^, occurred before growth of *Nitrospira,* but it has to be remembered that no absolute abundance were available. The increased mineralization might have resulted in the growth of heterotrophic copiotrophs, thereby reducing the relative abundance of AOB and NOB. As such, the relative abundance of AOB and NOB might have only increased when the growth of heterotrophic copiotrophs decreased, i.e. day 28 and 56.

### Soil predicted functional profile

Changes in organic material availability and composition will alter the bacterial community structure and might change its potential functionality^55^. The retention of crop residues together with tillage changes the available C and soil enzymatic activities and functional diversity^56^. Previous studies have shown that reduced or no tillage with crop residue retention increased the soil enzymatic and microbial functional activity related to C-compounds degradation, e.g. carbohydrate and phenolic compounds degradation and urease activity, compared to conventional tillage with or without crop residues in long-term arable soil experiments^57^. In this study, metabolic functions related to the degradation of complex C compounds and metabolism of N-compounds (assimilation and transportation) were enriched by tillage-residue management, but less than by the application of ammonium. Li et al.^58^ observed an enrichment of organic-N and N-compounds bacterial metabolism, e.g. nitrification, denitrification and assimilatory nitrate reduction, in a long-term N fertilization experiment.

## Conclusions

The soil bacterial community structure, diversity and its potential functionality was determined in an arable soil under two contrasting tillage systems (conventional tilled beds remade every year and permanent beds) with crop residue retained or burned and left unfertilized or fertilized with 300 kg urea-N ha^−1^. Irrespective of fertilization, the soil was N depleted. Application of NH_4_^+^ increased microbial activity and C mineralization as evidenced by an increase in emission of CO_2_ in both the fertilized and unfertilized soil. However, fertilizing the soil accelerated the oxidation of NH_4_^+^ and NO_2_^−^ independent of tillage or crop residue management. The soil bacterial structure was affected more by ammonium application than by fertilization in the field and tillage-residue management, i.e. CT, PBB or PBC. Application of ammonium had an immediate and strong effect on some bacterial groups. Well-known rhizospheric and/or endophytic bacteria with copiotrophic lifestyle, e.g. *Flavisolibacter*, *Pseudomonas*, *Rheinheimera*, *Pseudoxanthomonas*, were enriched. The C mineralization and N pathways related genes were strongly affected by ammonium application and to a lesser extent by N fertilizer and tillage-residue management. Tillage and crop residue management, and inorganic fertilizer application, i.e. N fertilizer application in the field and NH_4_^+^ addition in the laboratory had a strong effect on the bacterial community in this N depleted arable soil.

## Methods

### Study site and soil collection and characterization

Soil was collected in April 2012, from a long-term experiment initiated in 1992 with different agricultural practices at CIMMYT’s Norman E. Borlaug experimental station (CENEB) near Ciudad Obregon, Sonora, Mexico (lat. 27.33°N, long. 109.09°W, 38 m.a.s.l.). The six treatments were: conventional tilled beds with the crop residue retained and incorporated through tillage (considered the CT treatment), permanent beds with crop residue burned (considered the PBB treatment), and permanent beds with crop residue retained (considered the PBC treatment) fertilized with 300 kg urea-N ha^−1^ for wheat or not receiving N fertilization. The summer maize received a uniform N application of 150 kg N ha^−1^ in the first years of the experiment. From the summer of 2008 on, no N was applied to the maize in the treatments without N fertilization for wheat, while 150 kg N ha^−1^ was applied to the rest of the maize. All treatments received 46 kg P_2_O_5_ ha^−1^ for wheat and 50 kg P_2_O_5_ ha^−1^ for maize. The site has an arid climate and a mean annual temperature of 24.7 °C. Rainfall is dominant in the summer, with an average rainfall of 384 mm. Wheat (winter planted crop) and maize (summer planted crop) in annual rotation are irrigated in the furrows between the beds. Details of the field experimental design can be found in Verhulst et al.^6^.

Twenty subsamples (500 g) of the upper 0–15 cm soil layer were collected at random from three plots (*n* = 3) of each treatment (*n* = 6). The bulk soil was sampled at the tillering stage of maize. The soil sampled from each plot was pooled separately so that 18 composite samples were obtained (*n* = 18) (Fig. S10a). The field-based replication was maintained in the laboratory experiment to avoid pseudo-replication^59^. The soil samples were left to dry in the greenhouse, homogenized and sieved (2 mm mesh size). The particle size distribution, total C content, water holding capacity (WHC), moisture content, pH, electrolytic conductivity (EC), and mineral N (NH_4_^+^, NO_2_^−^ and NO_3_^−^) were determined (Fig. S10b). Details of the physical–chemical characterization of the soil can be found in Patiño-Zúñiga et al.^60^.

### Microcosms set up and experimental design

Sixteen 50 g sub-samples of soil (*n* = 6) from each plot (*n* = 3) were added to 120 mL flasks and adjusted to 40% WHC. The flasks were placed separately in a 1 L jar that contained a 25 mL flask with 20 mL 1 M NaOH to capture emitted CO_2_^61^. As such, a total of 288 experimental units were used in the study. The 1 L jars were closed air-tight and pre-incubated for 7 days. The soil was pre-incubated to eliminate any possible effect of handling the soil and adjust its water content^62^. The jars were opened and the 25 ml flask with 20 mL 1 M NaOH replaced. Half of the sixteen 50 g soil samples were amended with 1 mL of a 0.5 M solution of ammonium sulfate so that 300 mg N kg^−1^ dry soil was added to soil (considered the amended soil) and the soil water content was 50% WHC. The other half was amended with distilled water so that the water content was 50% of WHC (considered the unamended soil). The soil of both treatments was mixed. A flask from each plot (*n* = 18) and amended with NH_4_^+^ or left unamended was selected at random and 20 g soil extracted for mineral N with 80 ml 0.5 M K_2_SO_4_ filtered through a Whatman® No 41 filter paper and the mineral N in the extract determined on a Skalar autoanalyzer (Breda, The Netherlands)^63^. A 10 g sub-sample of soil was extracted for DNA and stored at − 21 °C pending analysis. These were considered zero time samples.

The jars were closed air-tight and incubated at 25 ± 2 °C for 56 days. After 1, 3, 5, 7, 14, 28 and 56 days, a flask with soil from each plot (*n* = 18) and amended with NH_4_^+^ or left unamended was selected at random (Fig. S11). The flasks were opened, the flask with NaOH removed and analyzed for the CO_2_ trapped^61^. The soil was removed from the flasks and part of it was extracted for DNA and the rest used to determine mineral N as described earlier.

### DNA extraction and 16S rRNA gene amplification and sequencing

Metagenomic DNA was extracted from soil using three methods that rely on mechanical and/or chemical/enzymatic lysis^64–66^. The first technique used the Hoffman and Winston lysis solution to lyse the microbial cells^64^, the second used lysozyme to lyse the cell walls based on the method developed by Sambrook and Russell^65^, and the third used a thermo/mechanical technique based on the method developed by Valenzuela-Encinas et al.^66^. The soil was homogenized using a FastPrep24 high-speed benchtop homogenizer (MP Biomedicals, Solon, OH, USA) at 4 m s^−1^. The three techniques were used to extract DNA twice from 0.5 mg soil samples from the three plots of each of the six treatments (*n* = 18), and then pooled to create a metagenomic sample. As such, a total of 3 g soil was extracted per plot, i.e. three extraction techniques applied twice to a 0.5 g soil subsample.

The V3-4 hypervariable region of the 16S rRNA gene (about 490 bp amplicon size) was amplified using 8-bp fused barcode primers 341-F (5′-CCTACGGGIGGCWGCAG-3′) and 805-R (5′-GACTACHVGGGTATCTAATCC-3′)^67^ with a two-step PCR protocol “16S metagenomic sequencing library preparation” published by Illumina Inc (15044223 Rev. B). Triplicate PCR amplification reactions per metagenomic DNA were done in a MultiGene OptiMax thermal cycler (Labnet International Inc.) under conditions previously reported^19^. The PCR amplification conditions were as follows: initial denaturation at 95 °C for 10 min, followed by 25 cycles of denaturation at 95 °C for 45 s, annealing at 53 °C for 45 s, extension at 72 °C for 1 min and a final extension at 72 °C for 10 min. A no-template control (negative PCR reaction) was included each time a PCR was done. The triplicate PCR reactions (12.5 µL each) were pooled and cleaned using FastGene™ columns (Nippon Genetics, Co., Ltd). Pooled and cleaned PCR products were quantified using the Invitrogen’s PicroGreen^®^ assay with a NanoDrop™ 3300 fluorospectrometer (Thermo Fisher Scientific Inc., Suwanee, CA) and standardized at equal molar amounts for later sequencing. As such, a total of 288 metagenomic DNA samples, i.e. one DNA sample from each soil sample, were PCR-amplified and sequenced. Both library normalization and sequencing process was done by Macrogen Inc. (Seoul, Korea). Amplicon libraries were paired-end sequenced using the MiSeq v3 platform (2 × 300 cycle kit) at Macrogen Inc. (Seoul, Korea).

### 16S rRNA gene sequence analysis

The 16S rRNA gene sequences were analyzed using the “Quantitative insights into microbial ecology (QIIME v1.9)” platform^68^. Briefly, reads from the same samples were demultiplexed based on the 8-bp barcodes and then merged using fastq-join method (100 nt as the minimum overlap region, *-j* 100). Chimeric sequences were identified and removed using VSEARCH v2.8^69^. Joined paired-end chimeric-free sequences were clustered into operational taxonomic units (OTUs) at 97% similarity and taxonomy was assigned by aligning to QIIME’s default Greengenes v13_8 database using PyNAST. The OTUs assigned to Archaea were discarded. The functional prediction of the nitrogen cycling related genes was assessed with PICRUSt v1.1^70^ using a down-sampled OTU-table to 3000 sequences per sample and using the general “cluster of orthologous groups of genes” (COGs) categorization. The PICRUSt analysis was done according to the “metagenome prediction tutorial”. The standard procedure included (i) normalization of the closed reference OTU table using the *normalize_by_copy_number.py* script and (ii) the functional predictions with *predict_metagenomes.py* script (using “cog” as the option for the *-type_of_prediction*). The selected COG identifiers related to nitrogen cycling were obtained from Li et al.^71^. Their relative abundance was calculated based on the total sum scaling per sample. The functional annotation of prokaryotic taxa (FAPROTAX, v.1.2) was used to investigate and map the functional profiles of soil bacterial communities^72^. The relative abundance of each functional role was determined based on the total sum scaling procedure within samples, and modules not related to environmental samples were ignored.

Rarefaction curves were made after pooling sequences of all individual soil samples from the same field treatment (*n* = 6), i.e., CT urea-fertilized (CT300) or unfertilized (CT0), PBB urea-fertilized (PBB300) or unfertilized (PBB0), and PBC urea-fertilized (PBC300) or unfertilized (PBC0). Sequences were pooled using both the *collapse_samples.py* and *alpha_rarefaction.py* scripts within QIIMEv1.9^68^ as described in “http://qiime.org/scripts/collapse_samples.html.”

The soil microbial diversity was determined with the Hill numbers at different *q* orders (*q* = 0, 1 and 2) using the raw count dataset at the genus taxonomic level as described by Ma and Li^73^. Hill numbers have the advantage over commonly used diversity indices that they maintain the same measurement unit across values, i.e. effective number of species, overcoming the bias due to rare and dominant species and they are comparable with the traditional diversity indices. At higher *q* orders, diversity values are more sensitive to common species^73^.

### Statistical analyses

All statistical analyses were done in R 3.6^74^. An ANOVA test (aov function in “stats” package) was used to determine the effect of the tillage-residue management (permanent beds with residue retained and burned and conventional beds), in-field fertilizer application (0 or 300 kg urea-N ha^−1^), and ammonium applied in the lab (unamended or soil amended with 300 mg NH_4_^+^-N kg^−1^ soil) on emitted CO_2_ and mineral N after 56 days. The Tukey-HSD test using all pair-wise comparison, i.e. *post*-*hoc*, was done with the “agricolae” package (HSD.test function)^75^. A non-parametric test (aldex.kw function; Kruskal Wallis test) in the ALDEx2 package^14^ was used to determine the effect of the treatments on the different bacterial groups using the centered-log-ratio transformed counts, i.e. clr-transformation. The clr-transformation was done using the ALDEx2 (v1.18) package as high-throughput data are compositional^14,76^. A principal component analysis (PCA) was done using the clr-transformed sequence counts to explore the effect of tillage-residue management, fertilizer application, and ammonium applied on the bacterial community structure. A preliminary PCA analysis grouped the bacterial communities of day 0, 1 and 3 together, those of day 5, 7 and 14, and those of day 28 and 56. To facilitate our analysis and better visualize this, similar colours were given to these groups in the PCA graphs. The permutational multivariate analyses of variance (perMANOVA) test was used to determine the effect of tillage-residue management, fertilizer application, ammonium applied and incubation time on the bacterial community structure. The perMANOVA was done using the clr-transformed sequence counts for taxonomic and functional data. Time (days) was embedded in the perMANOVA analysis using “strata” argument when tillage-residue management and fertilizer application or ammonium application were used as fixed factors within adonis function. The PCA and perMANOVA tests were done with the FactoMineR^77^ and vegan packages^78^. Heatmaps were constructed with the pheatmap package^79^.

The effect of ammonium application on the bacterial groups was calculated by a ratio in two different ways.

First, when the relative abundance of the bacterial group was higher in the ammonium amended soil than in the unamended soil then the ratio was calculated as:

$$ {\text{Ratio }} = \, \left( {{\text{relative abundance of the bacterial group in the NH}}_{{4}}^{ + } {\text{amended soil }} - {\text{ relative abundance of the bacterial group in the unamended soil}}} \right)/\left( {\text{relative abundance of the bacterial group in the unamended soil}} \right). $$

Second, when the relative abundance of the bacterial group was lower in the ammonium amended soil than in the unamended soil then the ratio was calculated as:

$$ {\text{Ratio }} = \, \left( {{\text{relative abundance of the bacterial group in the unamended soil }} - {\text{ relative abundance of the bacterial group in the NH}}_{{4}}^{ + } {\text{amended soil}}} \right)/\left( {{\text{relative abundance of the bacterial group in the NH}}_{{4}}^{ + } {\text{amended soil}}} \right). $$

## Supplementary Information

Supplementary Information.

## Data Availability

The raw sequenced datasets were deposited in the GenBank database under the BioProject accession number PRJNA545497. Any additional data will be provided upon request.
