# Supplementary material for: Application of ammonium to a N limited arable soil enriches a succession of bacteria typically found in the rhizosphere
Source: Sci Rep. 2022 Mar 8;12:4110. doi: 10.1038/s41598-022-07623-4 (PMC8904580; doi:10.1038/s41598-022-07623-4)
Supplement: Supplementary file 1 — Supplementary Information. [file 41598_2022_7623_MOESM1_ESM.pdf]

Supplementary Material

Application of ammonium to a N limited arable soil enriches a succession of bacteria typically found in the rhizosphere

Mario Hernández-Guzmán [1], Valentín Pérez-Hernández [1, 2], Yendi E. Navarro-Noya [3], Marco L. Luna-Guido [1], Nele Verhulst [4], Bram Govaerts [4, 5], Luc Dendooven [1*]

[1] Laboratory of Soil Ecology, CINVESTAV, Mexico city, Mexico,

[2] Department of Chemistry and Biochemistry, Instituto Tecnológico de Tuxtla-Gutiérrez, Tuxtla Gutiérrez, Mexico,

[3] Centro de Investigación en Ciencias Biológicas, Universidad Autónoma de Tlaxcala, México,

[4] International Maize and Wheat Improvement Center (CIMMYT), El Batán, Texcoco, Mexico,

[5] Cornell University, Ithaca, USA.

* Corresponding author: Luc Dendooven, Av. Instituto Politécnico Nacional 2508, Col. San Pedro Zacatenco, Alcaldía Gustavo A Madero, Mexico city, Mexico. Tel.: +52 5550613319, +52 5550613800, *e-mail*: dendooven@me.com

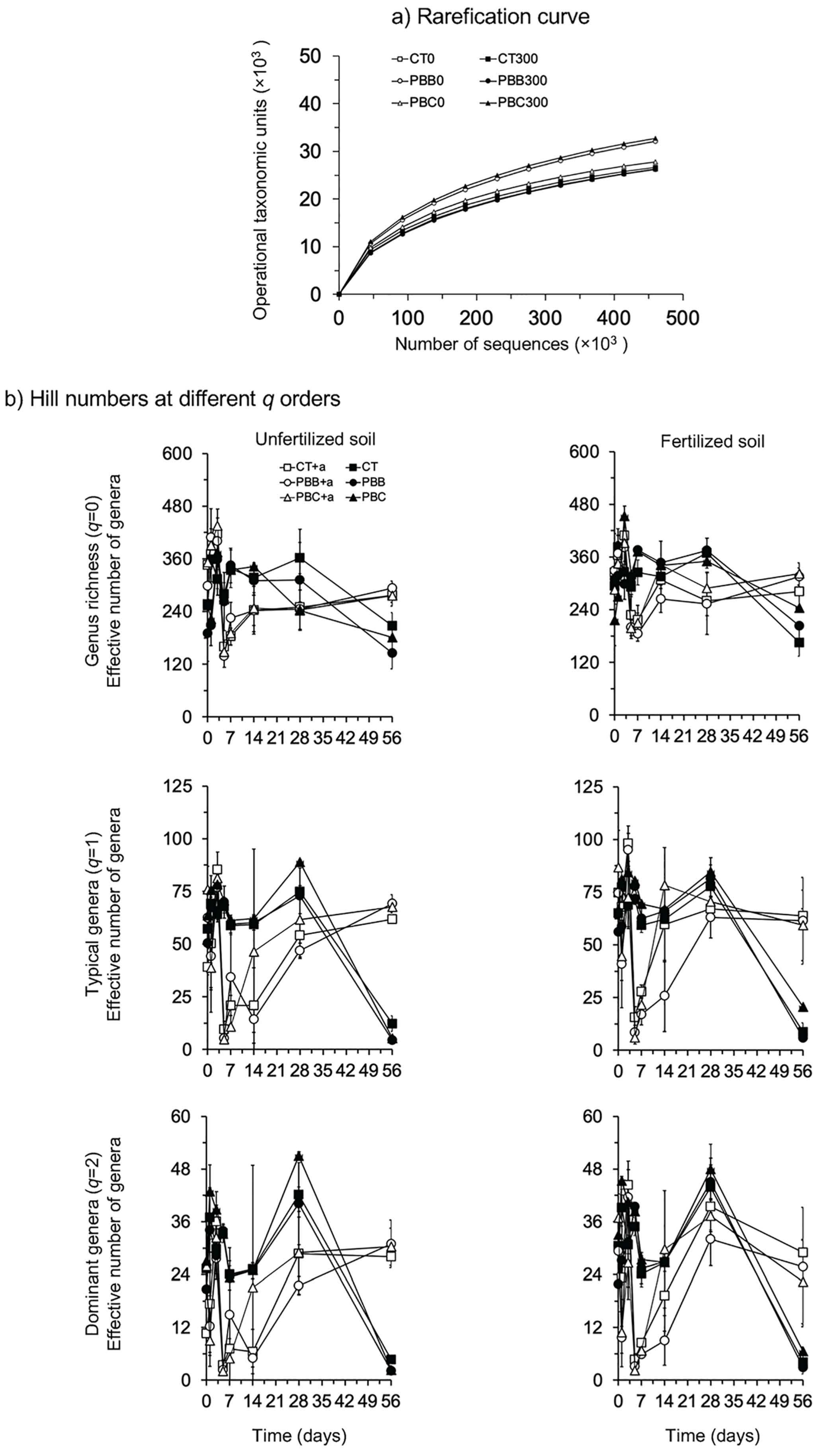

Supplementary Fig S1. a) Rarefication curves with soil with conventional tilled beds, crop residue retained left unfertilized (CT, □) or fertilized with 300 kg urea-N ha^{-1} (■), permanent beds with crop residue burned left unfertilized (PBB, ○) or fertilized with 300 kg urea-N ha^{-1} (●), permanent beds with crop residue retained left unfertilized (PBC, △) or fertilized with 300 kg urea-N ha^{-1} (▲). b) Hill numbers at different *q* orders with species richness at $q = 0$, typical genera at $q = 1$, and dominant genera ($q = 2$) in CT soil left unamended (■) or amended with 300 mg NH_4^+-N ha^{-1} (CT+a, □), PBB soil left unamended (●) or amended with 300 mg NH_4^+-N ha^{-1} (PBB+a, ○) and PBC soil left unamended (▲) or amended with 300 mg NH_4^+-N ha^{-1} (PBC+a, △).

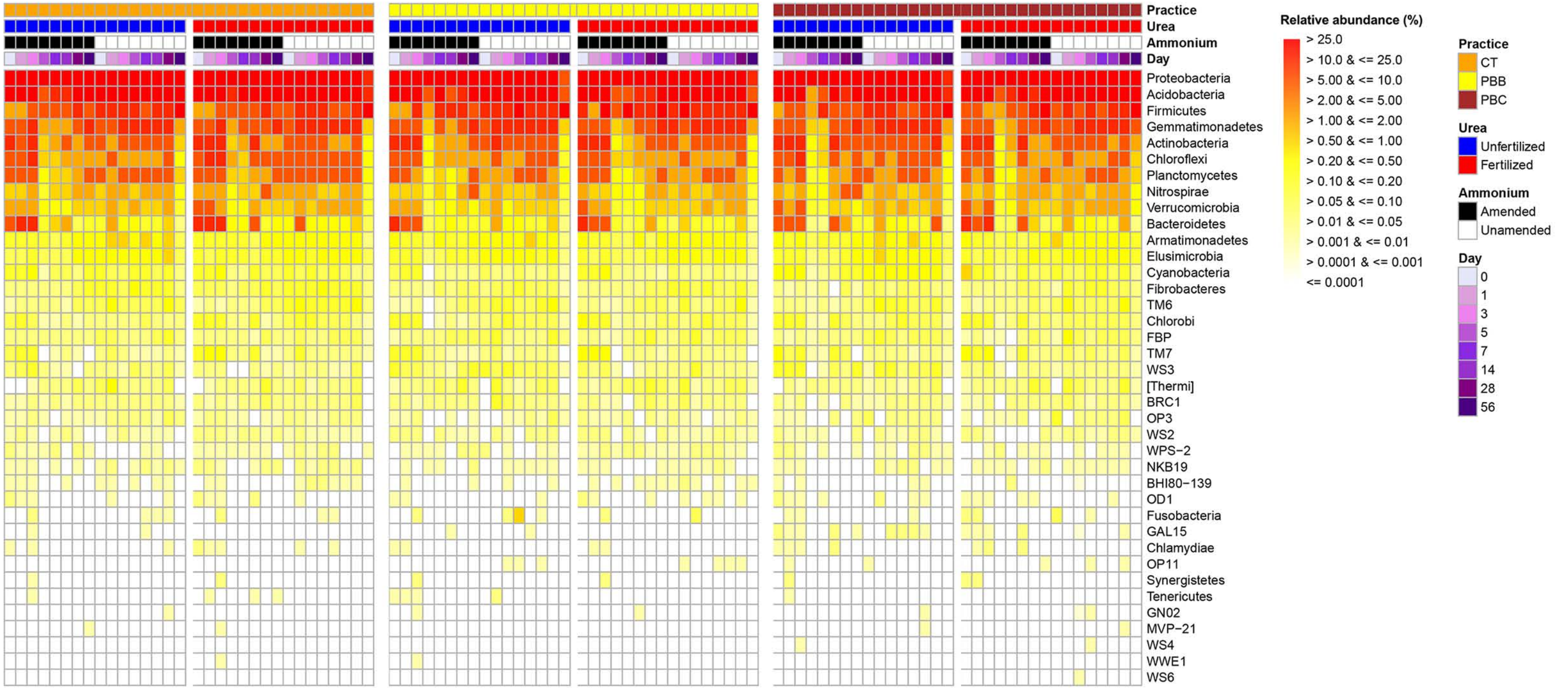

Supplementary Fig. S2. Heat map with the relative abundance of the bacterial phyla in the Norman E. Borlaug experimental station (CENEB) soil incubated aerobically for 56 days.

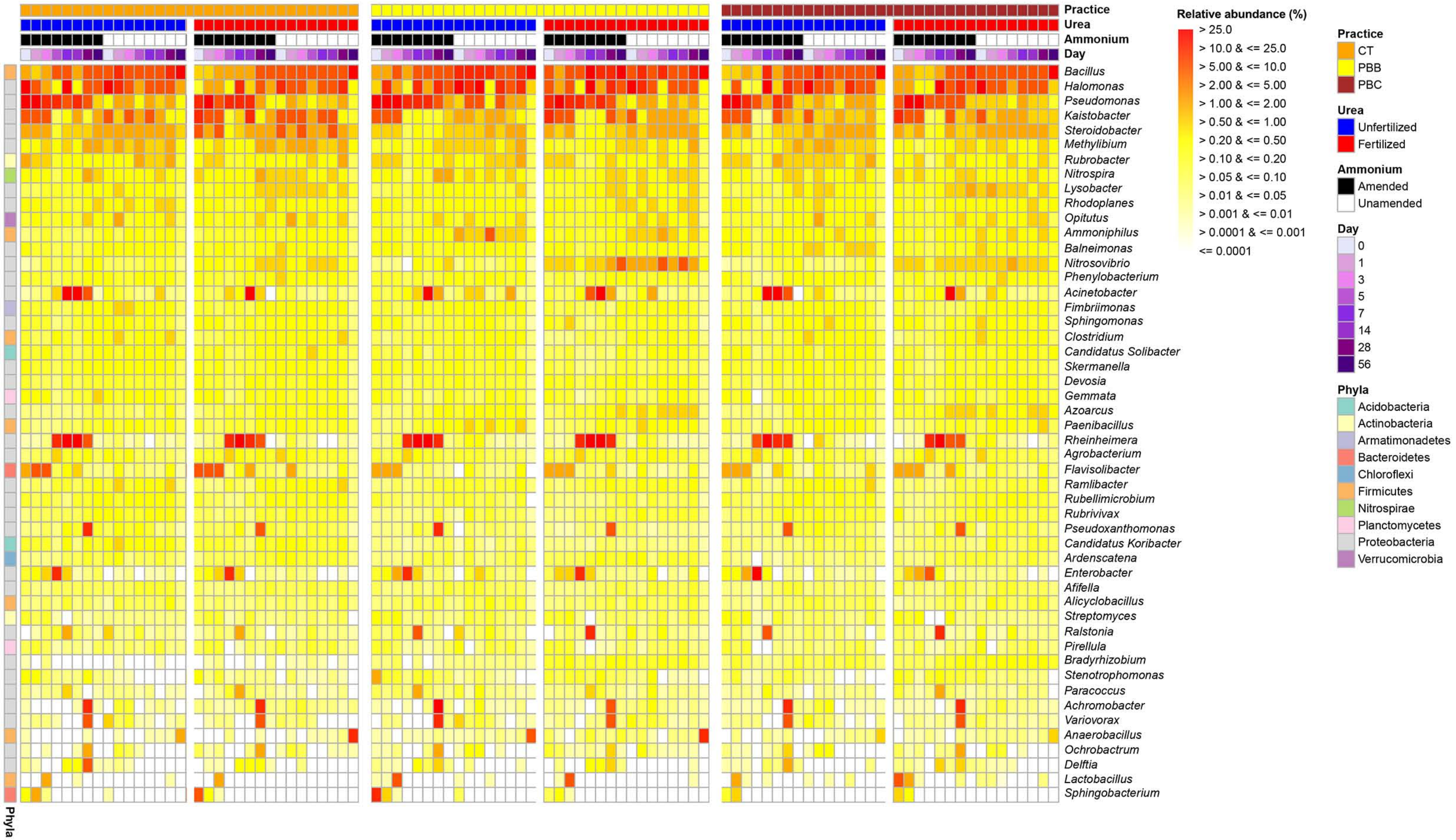

Supplementary Fig. S3. Heat map with the relative abundance of the 50 most abundant bacterial genera in the Norman E. Borlaug experimental station (CENEB) soil incubated aerobically for 56 days.

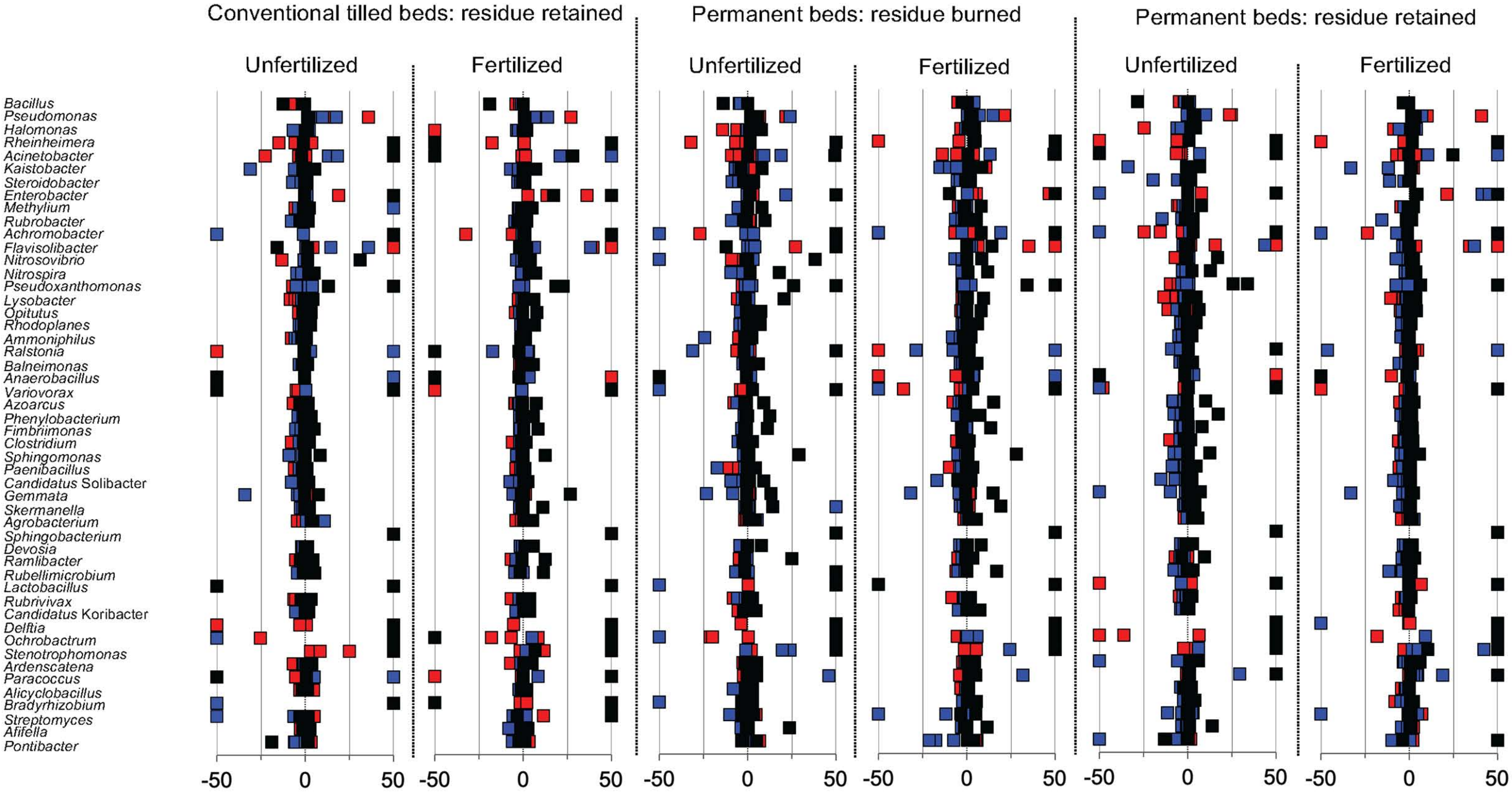

Supplementary Fig. S4. Ratio of the relative abundances of the 50 most abundant bacterial genera in the soil amended with 300 mg NH_4^+-N kg^{-1} versus the unamended soil in the unfertilized soil or soil fertilized with 300 kg urea-N ha^{-1} with conventional tilled beds (CT), permanent beds with crop residue burned (PBB) and permanent beds with crop residue retained (PBC) at day 0, 1 and 3 (■), at day 5, 7 and 14 (■) and at day 28 and 56 (■) incubated aerobically at 25±2°C.

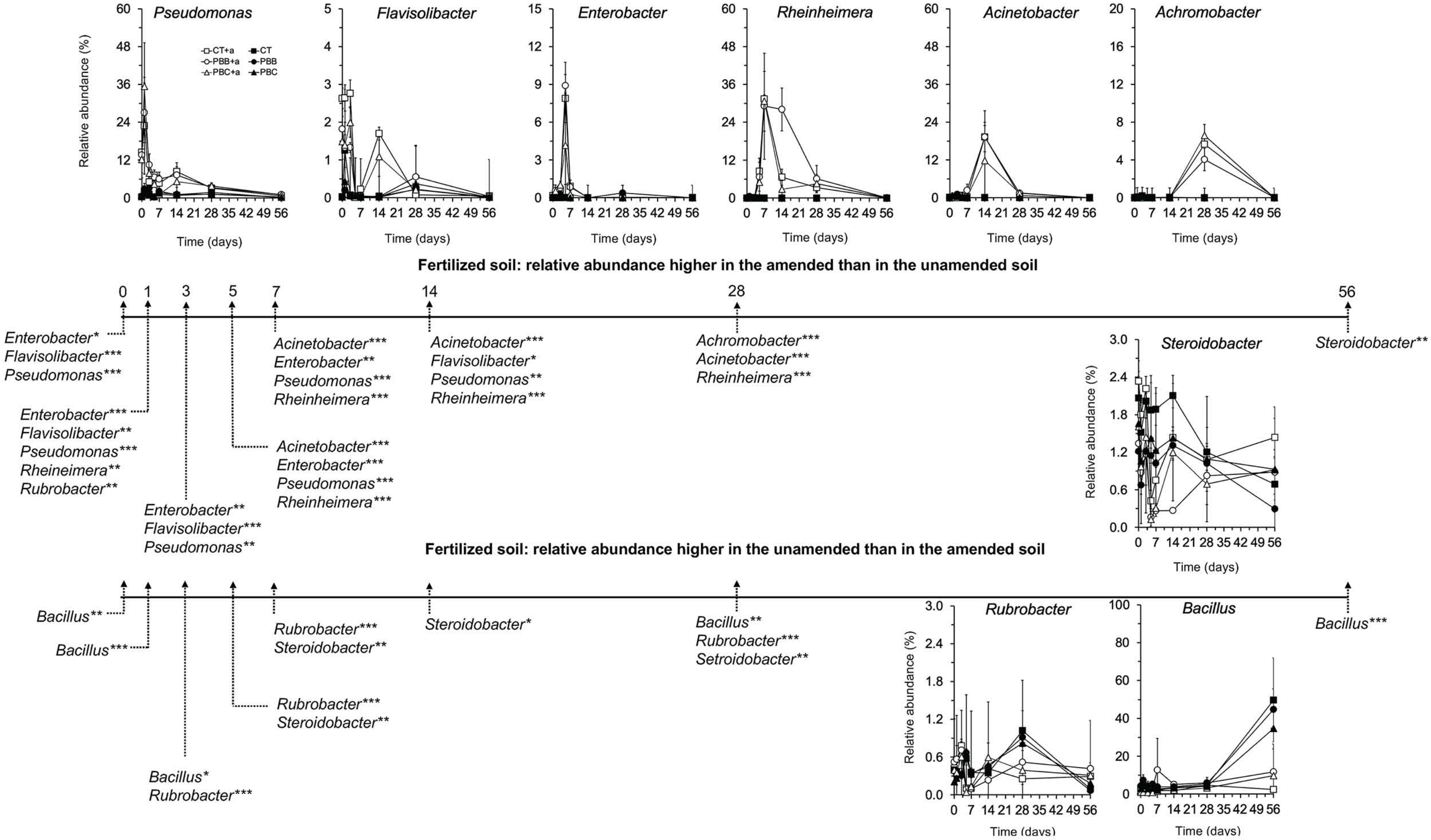

Supplementary Fig. S5. Changes in the relative abundance (%) of bacterial genera in the soil fertilized with 300 kg urea-N ha^{-1} with conventional tilled beds and crop residue retained (CT) left unamended (■) or amended with 300 mg NH_4^+-N (□), permanent beds with crop residue burned (PBB) left unamended (●) or amended with 300 mg NH_4^+-N (○) and permanent beds with crop retained incubated left unamended (▲) or unamended with 300 mg NH_4^+-N (△) incubated aerobically at 25±2°C for 56 days. A non-parametric test (aldex.kw function; Kruskal Wallis test) in the ALDEx2 package [1] was used to determine the effect of application of 300 mg NH_4^+-N on the relative abundance of the bacterial genus using the centered-log-ratio transformed counts, i.e. clr-transformation, on the different sampling days with *** $P < 0.001$, ** $P < 0.01$ and $P \geq 0.001$, and * $P < 0.05$ and $P \geq 0.01$.

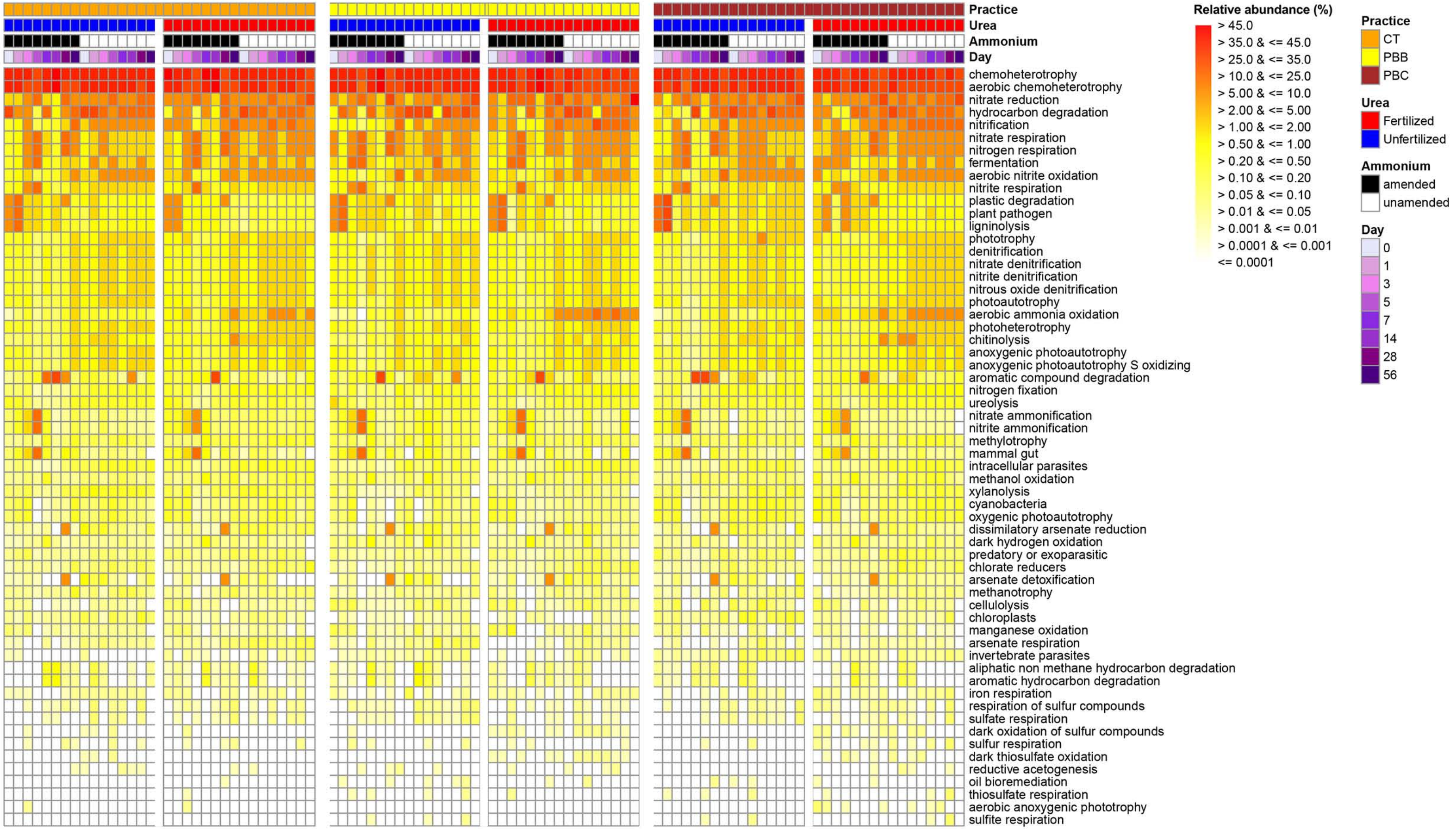

Supplementary Fig. S6. Heat map with the relative abundance of the functional annotation of prokaryotic taxa (FAPROTAX, v.1.2).

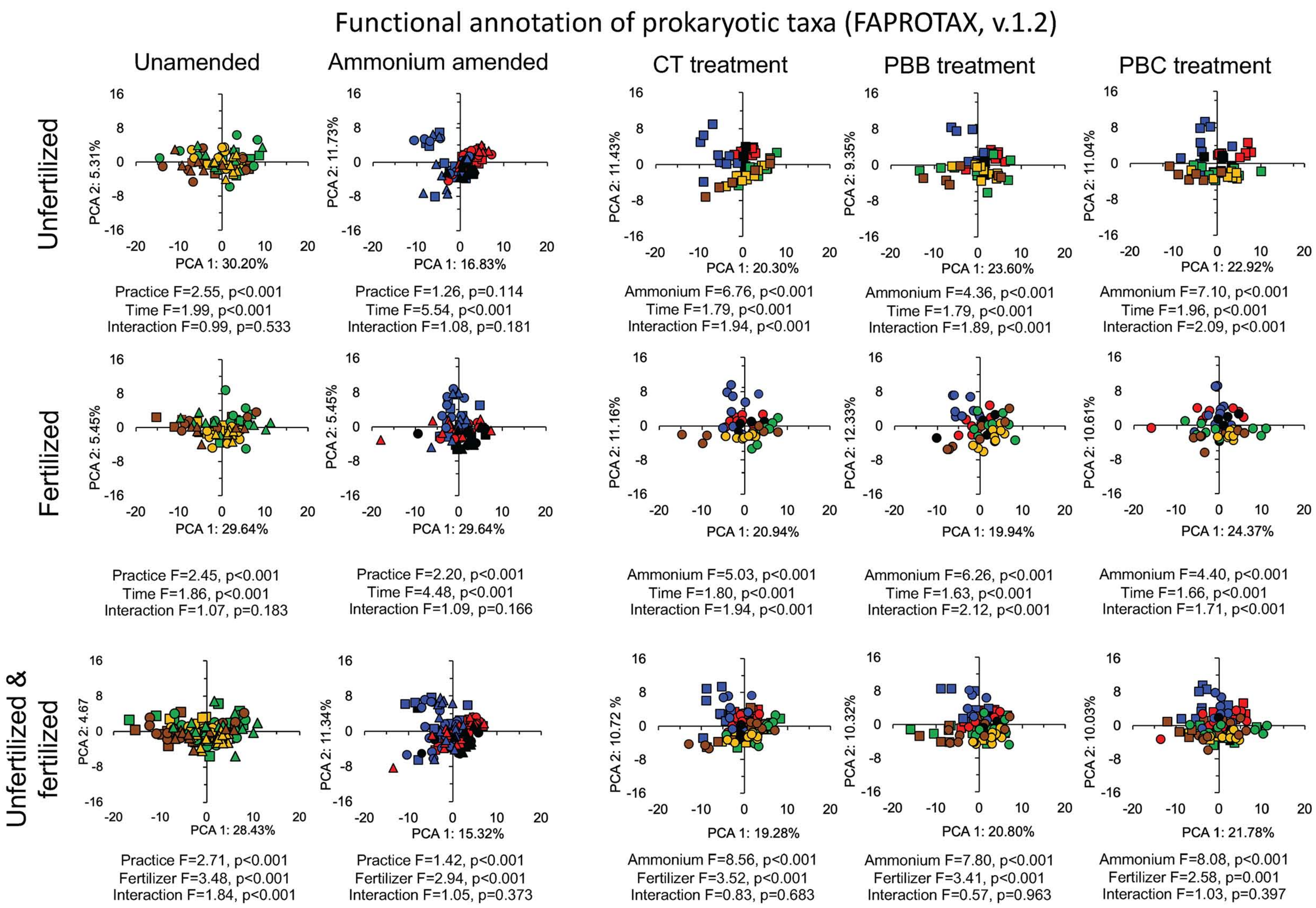

Supplementary Fig. S7. Principal component analysis (PCA) with the converted sequence counts of the functional annotation of prokaryotic taxa (FAPROTAX, v.1.2) using the centred log-ratio transformation (aldex.clr argument, ALDEx2 package, [2]) in the unfertilized soil or soil fertilized with 300 kg urea-N ha^{-1} left unamended or amended with 300 mg NH_4^+-N kg^{-1} with conventional tilled beds (CT), permanent beds with crop residue burned (PBB) and permanent beds with crop residue retained (PBC) incubated aerobically at 25±2°C for 56 days. F and p values were determined with a perMANOVA analysis. The legend to the Figure can be found in Fig. 3.

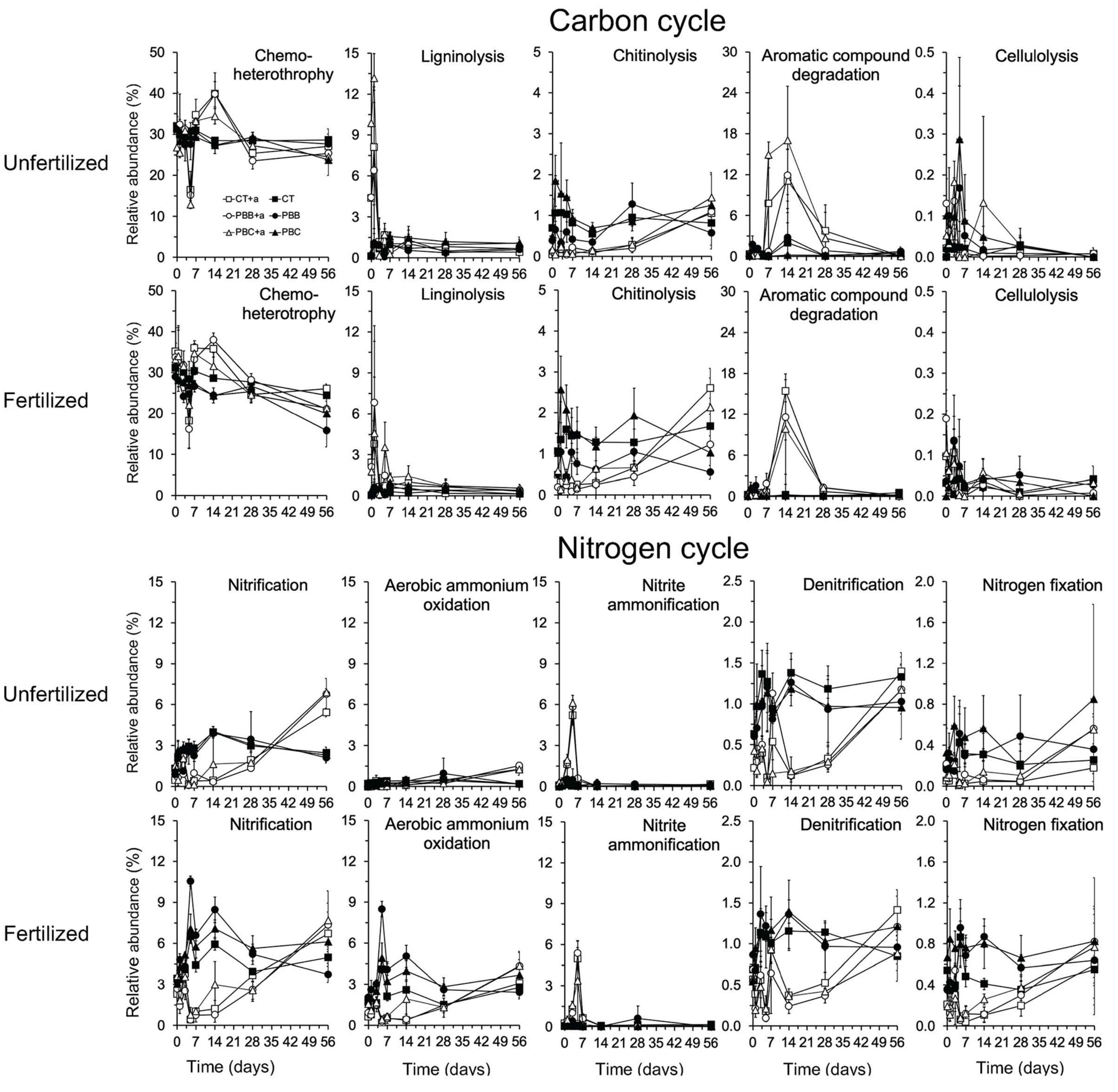

Supplementary Fig. S8. Dynamics of some selected functional annotation of prokaryotic taxa of the carbon and nitrogen cycle (FAPROTAX, v.1.2) in the unfertilized soil and soil fertilized with 300 kg urea-N ha^{-1} with conventional tilled beds and crop residue retained (CT) left unamended (■) or amended with 300 mg NH_4^+-N (□), permanent beds with crop residue burned (PBB) left unamended (●) or amended with 300 mg NH_4^+-N (○) and permanent beds with crop retained incubated left unamended (▲) or amended with 300 mg NH_4^+-N (△) incubated aerobically at 25±2°C for 56 days.

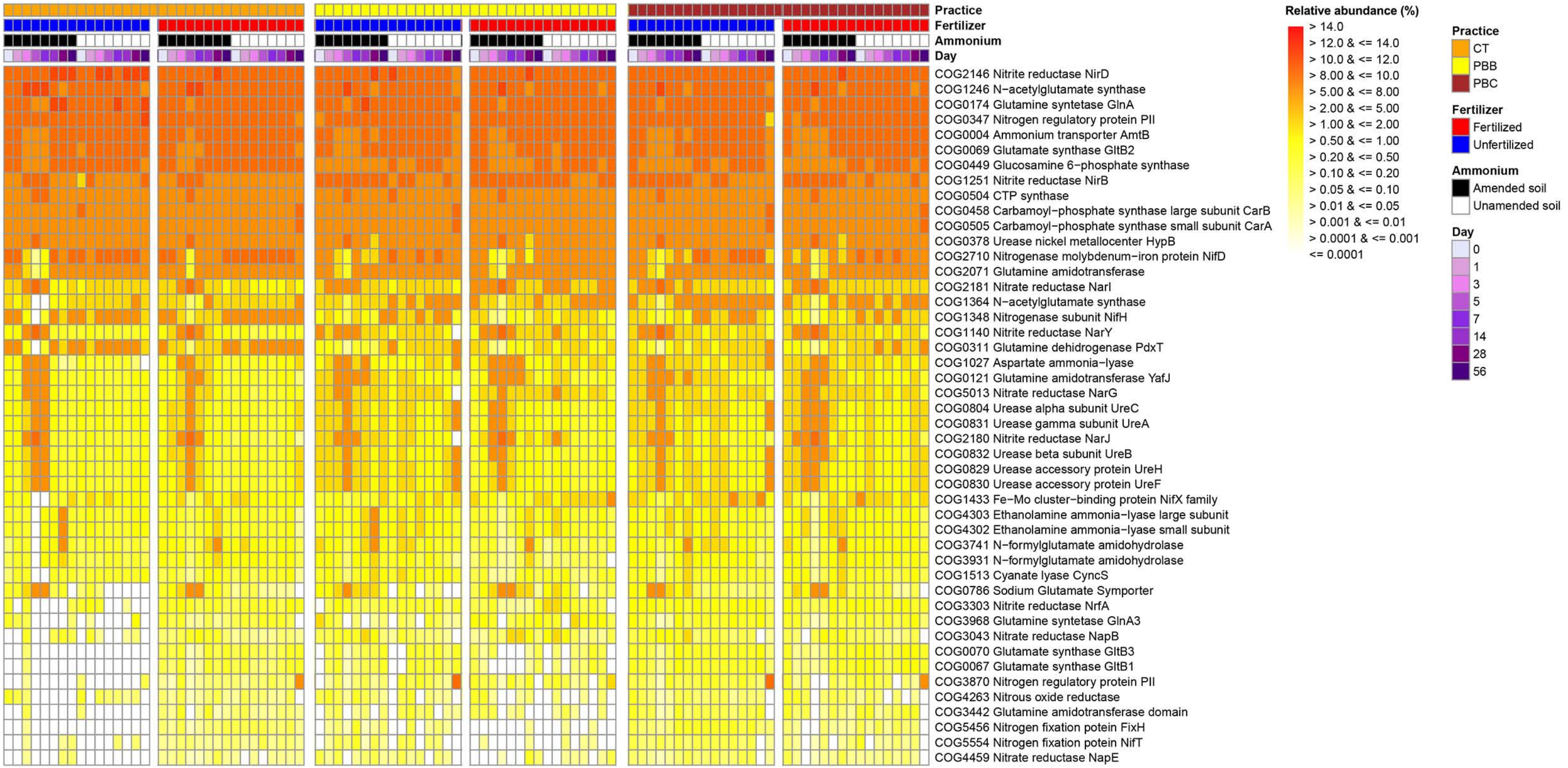

Supplementary Fig. S9. Heat map with the relative abundance of the relative abundance of N-cycle related genes based on the cluster of orthologous groups (COGs).

a) Soil sampling procedure

Each treatment sampled (n = 3) was fertilized with 300 kg urea-N ha^{-1} y^{-1} or left unfertilized since 1992

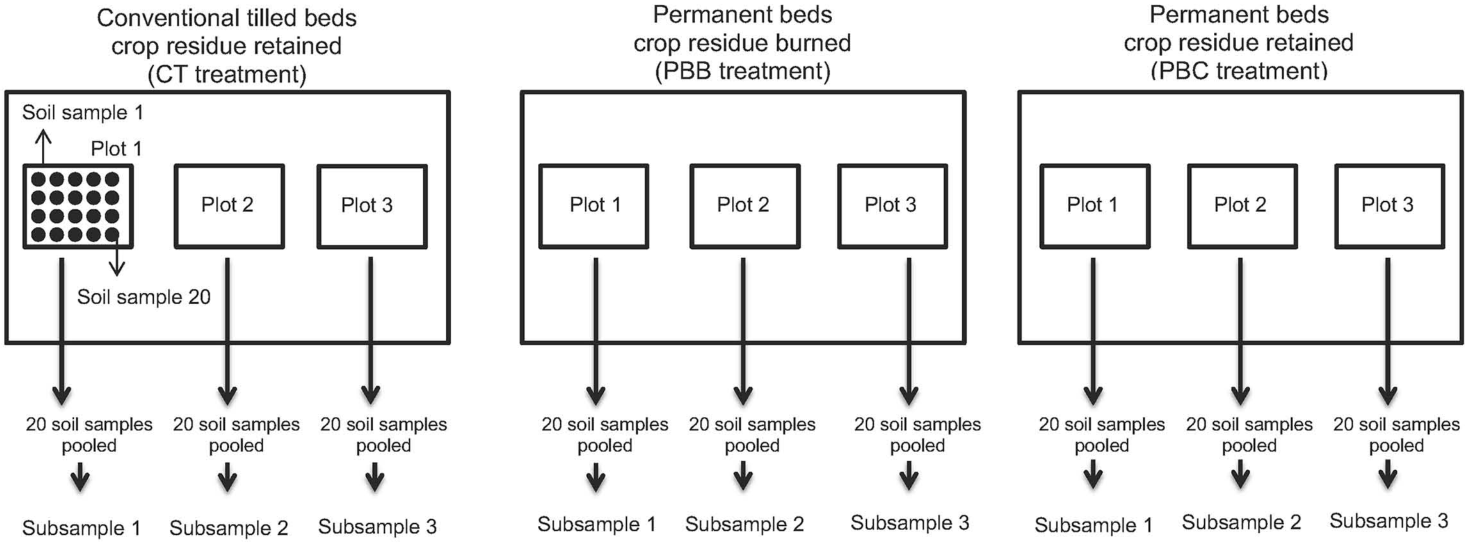

Soil of each subsample was left unamended or amended with 300 mg NH_4^+-N and incubated aerobically for 56 days.
Emission of CO_2, dynamics of mineral N and the bacterial community structure were determined.

b) Soil characteristics

Characteristics of soil with different agricultural practices left unfertilized or fertilized with urea at the CIMMYT's Norman E. Borlaug (CENEB) experimental station (Sonora, Mexico).

Agricultural practice	Urea (kg N ha^{-1} y^{-1})	pH	EC [a] (dS m^{-1})	WHC [b]	C_{tot} [c]	N_{tot} [d]	C/N ratio	Sand	Silt	Clay
				(g kg^{-1} soil)				(g kg^{-1} soil)		
CT	0	7.9 [e] ± 0.2 [f]	1.18 ± 0.29	791 ± 36	9.3 ± 2.4	0.60 ± 0.05	15.5	720 ± 30	270 ± 40	10 ± 10
	300	7.9 ± 0.2	1.67 ± 0.54	824 ± 24	11.9 ± 1.3	0.83 ± 0.13	14.3	700 ± 40	280 ± 50	20 ± 10
PBB	0	8.1 ± 0.2	1.94 ± 0.04	841 ± 30	5.7 ± 1.9	0.49 ± 0.03	11.6	580 ± 50	330 ± 40	90 ± 10
	300	7.7 ± 0.2	1.50 ± 0.48	793 ± 19	9.4 ± 3.2	0.76 ± 0.17	12.4	690 ± 60	280 ± 50	30 ± 10
PBC	0	8.1 ± 0.4	1.06 ± 0.24	821 ± 19	11.3 ± 5.4	0.56 ± 0.03	20.2	630 ± 110	280 ± 50	90 ± 60
	300	7.9 ± 0.1	1.18 ± 0.16	796 ± 26	9.6 ± 1.5	0.67 ± 0.11	14.3	700 ± 50	260 ± 30	30 ± 20

[a] EC: Electrolytic conductivity, [b] WHC: Water holding capacity, [c] C_{tot}: Total carbon, [d] N_{tot}: Total nitrogen, [e] Mean of three replicate plots, [f] Standard deviation of the mean.

Supplementary Fig. S10. Soil sampling and soil characteristics at CIMMYT's Norman E. Borlaug experimental station (CENEB).

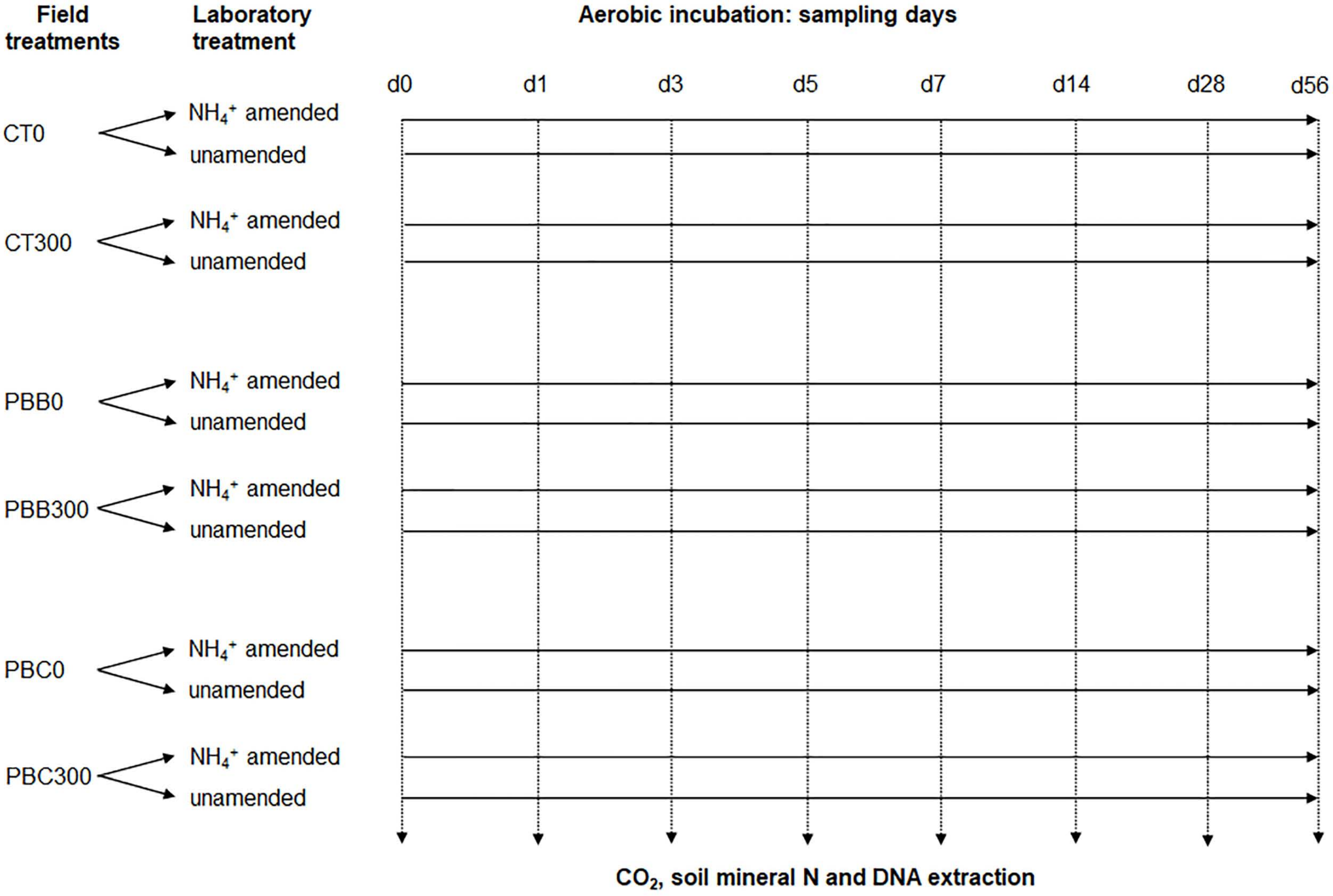

Supplementary Fig. S11. Experimental Design.

TABLE S1. Effect of agricultural practices (CT, permanent beds conventional till, PBB, permanent beds with residue burn or PBC, permanent beds with residue kept) on bacterial phyla and bacterial groups assigned up to the level of genus in the unamended soil and soil amended with 300 mg NH_4^+-N kg^{-1} using a compositional approach, i.e. analysis of differential abundance taking sample variation into account with the aldex.kw argument (ALDEx2 package, [2]).

Unfertilized soil				Soil fertilized with 300 kg urea-N ha^{-1}			
Unamended soil		Amended soil		Unamended soil		Amended soil	
Bacterial group	*P* value	Bacterial group	*P* value	Bacterial group	*P* value	Bacterial group	*P* value
Cyanobacteria [a]	8.21×10^{-5} [b]			Cyanobacteria	2.72×10^{-2}		
Fibrobacteres	1.78×10^{-3}			WS3	3.79×10^{-2}		
Candidatus Koribacter	5.16×10^{-7}	*Candidatus* Koribacter	1.11×10^{-3}	*Bradyrhizobium*	5.66×10^{-8}	*Bradyrhizobium*	6.93×10^{-6}
Rubellimicrobium	2.96×10^{-5}	*Ammoniphilus*	3.49×10^{-3}	*Nitrosovibrio*	6.09×10^{-6}	*Candidatus* Koribacter	7.82×10^{-3}
Rubrivivax	4.02×10^{-5}	*Bradyrhizobium*	2.29×10^{-2}	*Candidatus* Koribacter	3.33×10^{-5}	*Sporosarcina*	8.53×10^{-3}
Steroidobacter	3.93×10^{-4}	*Sphingobium*	1.11×10^{-3}	*Phaeospirillum*	2.86×10^{-4}	*Steroidobacter*	1.14×10^{-2}
Bradyrhizobium	6.38×10^{-4}			*Ammoniphilus*	4.97×10^{-4}	*Sinorhizobium*	2.04×10^{-2}
Ammoniphilus	1.46×10^{-3}			*Pontibacter*	6.29×10^{-4}	*Brevundimonas*	2.10×10^{-2}
Methylibium	2.51×10^{-3}			*Steroidobacter*	1.29×10^{-3}	*Pontibacter*	2.15×10^{-2}
Rhizobium	1.46×10^{-2}			*Azohydromonas*	2.26×10^{-3}	*Acidovorax*	2.84×10^{-2}
Hyphomicrobium	1.72×10^{-2}			*Skermanella*	5.79×10^{-3}	*Nitrosovibrio*	4.73×10^{-2}
Kaistobacter	1.95×10^{-2}			*Methylibium*	6.92×10^{-3}		
Fimbriimonas	2.71×10^{-2}			*Pseudoxanthomonas*	1.21×10^{-2}		
Sphingobium	2.94×10^{-2}			*Rubellimicrobium*	2.12×10^{-2}		
Bacillus	3.02×10^{-2}			*Brevibacillus*	2.19×10^{-2}		
Candidatus Solibacter	3.20×10^{-2}			*Ramlibacter*	2.52×10^{-2}		
Gemmata	3.44×10^{-2}			*Bacillus*	2.59×10^{-2}		
Rhodoplanes	3.77×10^{-2}			*Sporosarcina*	3.18×10^{-2}		
Myxococcus	4.01×10^{-2}			*Caloramator*	3.79×10^{-2}		
Afifella	4.29×10^{-2}			*Anoxybacillus*	4.73×10^{-2}		
Ardenscatena	4.71×10^{-2}						

[a] Only the bacterial phyla and the groups assigned up to the taxonomic level of genus significantly affected by agricultural practice $P < 0.05$, [b] The expected values of the Kruskal-Wallis test for each feature obtained with aldex.kw argument with converted sequence data using the centred log-ratio transform test returned by the aldex.clr argument ALDEx2 package.

References to the Supplementary Material

1. G. B. Gloor, J. M. Macklaim, V. Pawlowsky-Glahn, J. J. Egozcue, Microbiome datasets are compositional: And this is not optional. *Front. Microbiol.* **8** (2017).
2. G. B. Gloor, A. D. Fernandes, J. M. Macklaim, Analysis of Differential Abundance Taking Sample Variation into Account: Package “ALDEx2”. 2019. *https://Github.Com/Ggloor/ALDEx_bioc*.
